# Genome-Wide Identification by Transposon Insertion Sequencing of Escherichia coli K1 Genes Essential for *In Vitro* Growth, Gastrointestinal Colonizing Capacity, and Survival in Serum

**DOI:** 10.1128/JB.00698-17

**Published:** 2018-03-12

**Authors:** Alex J. McCarthy, Richard A. Stabler, Peter W. Taylor

**Affiliations:** a School of Pharmacy, University College London, London, United Kingdom; b London School of Hygiene and Tropical Medicine, London, United Kingdom; Princeton University

**Keywords:** *Escherichia coli*, essential genes, neonates, rat infection model, transposon sequencing

## Abstract

Escherichia coli K1 strains are major causative agents of invasive disease of newborn infants. The age dependency of infection can be reproduced in neonatal rats. Colonization of the small intestine following oral administration of K1 bacteria leads rapidly to invasion of the blood circulation; bacteria that avoid capture by the mesenteric lymphatic system and evade antibacterial mechanisms in the blood may disseminate to cause organ-specific infections such as meningitis. Some E. coli K1 surface constituents, in particular the polysialic acid capsule, are known to contribute to invasive potential, but a comprehensive picture of the factors that determine the fully virulent phenotype has not emerged so far. We constructed a library and constituent sublibraries of ∼775,000 Tn*5* transposon mutants of E. coli K1 strain A192PP and employed transposon-directed insertion site sequencing (TraDIS) to identify genes required for fitness for infection of 2-day-old rats. Transposon insertions were lacking in 357 genes following recovery on selective agar; these genes were considered essential for growth in nutrient-replete medium. Colonization of the midsection of the small intestine was facilitated by 167 E. coli K1 gene products. Restricted bacterial translocation across epithelial barriers precluded TraDIS analysis of gut-to-blood and blood-to-brain transits; 97 genes were required for survival in human serum. This study revealed that a large number of bacterial genes, many of which were not previously associated with systemic E. coli K1 infection, are required to realize full invasive potential.

**IMPORTANCE** Escherichia coli K1 strains cause life-threatening infections in newborn infants. They are acquired from the mother at birth and colonize the small intestine, from where they invade the blood and central nervous system. It is difficult to obtain information from acutely ill patients that sheds light on physiological and bacterial factors determining invasive disease. Key aspects of naturally occurring age-dependent human infection can be reproduced in neonatal rats. Here, we employ transposon-directed insertion site sequencing to identify genes essential for the *in vitro* growth of E. coli K1 and genes that contribute to the colonization of susceptible rats. The presence of bottlenecks to invasion of the blood and cerebrospinal compartments precluded insertion site sequencing analysis, but we identified genes for survival in serum.

## INTRODUCTION

Early-onset sepsis and associated septicemia and meningitis are major causes of morbidity and mortality in the first weeks of life. In the developed world, encapsulated Escherichia coli and group B streptococci are responsible for the large majority of these infections (1–3). Over 80% of E. coli blood and cerebrospinal fluid isolates from infected neonates express the α,2-8-linked polysialic acid (polySia) capsular K1 polysaccharide (4, 5), a polymer facilitating the evasion of neonatal immune defenses due to its structural similarity to the polySia modulator of neuronal plasticity in the developing human embryo (6). Infections arise due to colonization of the neonatal gastrointestinal (GI) tract by maternally derived E. coli K1 at or soon after birth, from where the bacteria invade the systemic circulation to gain entry to the central nervous system (CNS) (7, 8).

Essential features of human infection can be reproduced in the neonatal rat, enabling investigation of the pathogenesis of invasive neonatal infections by E. coli K1 (9–11). In susceptible 2-day-old (P2) rat pups, the protective mucus layer in the small intestine (SI) is poorly developed but matures to full thickness over the period from P2 to P9, coincident with the development of resistance to invasive infection from GI tract-colonizing E. coli K1 (12). Thus, oral administration of E. coli K1 initiates stable colonization of the small intestine in both P2 and P9 pups but elicits lethal systemic infection only in younger animals (13). In the absence of an effective mucus barrier at P2, the colonizing bacteria make contact with the apical surface of enterocytes in the midregion of the small intestine before translocation to the submucosa by an incompletely defined transcellular pathway (12). The bacteria subsequently gain access to the blood compartment by evading mesenteric lymphatic capture (10, 14). E. coli K1 cells strongly express polySia in blood (15), and the capsule may protect the bacteria from complement attack during this phase of infection by facilitating the binding of complement regulatory factor H to surface-bound C3b to prevent the activation of the alternative pathway (16, 17). Following hematogenous spread, the bacteria enter the CNS via the blood-cerebrospinal barrier at the choroid plexus epithelium to colonize the meninges (15). Some microorganisms that invade the CNS enter across the cerebral microvascular endothelium of the arachnoid membrane (18), although the restricted distribution of E. coli K1 within the CNS suggests that this is not a primary route of entry for this pathogen.

Only a limited number of pathogenic bacteria have the capacity to invade the CNS from a remote colonizing site, and the large majority elaborate a protective capsule that facilitates the avoidance of host defenses during transit to the site of infection (19). Although the polySia capsule is clearly necessary for the neonatal pathogenesis of E. coli K1 (11), the large majority of bacterial virulence factors that facilitate transit from the GI tract to the brain are unknown. A number of potential virulence factors associated with neonatal bacterial meningitis have been defined by phylogenetic analysis (20), and there is good evidence that the genotoxin colibactin and the siderophore yersiniabactin contribute to the pathogenesis of E. coli K1 in experimental rats (21–23); however, a more detailed understanding of virulence mechanisms of E. coli K1 invasive disease will present opportunities for new modes of therapy for these devastating infections.

Transposon insertion sequencing (24, 25), a combination of traditional transposon mutagenesis and massively parallel DNA sequencing, is a powerful tool for the genome-wide enhanced genetic screening of large pools of mutants in a single experiment. This method was recently used to determine the full complement of genes required for the expression of the K1 capsule by a uropathogenic E. coli isolate (26). This technique can be used to detect variations in the genetic fitness of individual mutants undergoing selection in colonized and infected hosts. There are a number of variations of this procedure, but they all rely on the creation of a pool of insertion mutants in which every locus has been disrupted at multiple sites; determination of the site of transposon insertion by sequencing of transposon junctions within chromosomal DNA before and after the application of selective pressure will identify mutants attenuated under selective conditions (27). Thus, genes that confer fitness during Klebsiella pneumoniae (28) and Acinetobacter baumannii (29) lung persistence, systemic and mucosal survival of Pseudomonas aeruginosa (30), and spleen colonization in the mouse by uropathogenic E. coli (31) have been identified by this approach. In this study, we employ transposon-directed insertion site sequencing (TraDIS) (24) to interrogate a library of ∼775,000 Tn*5* mutants or constituent sublibraries of E. coli K1 strain A192PP for genes essential for growth *in vitro* and for GI colonization, invasion, and systemic survival in susceptible P2 rat pups. In addition, we identified “bottlenecks” (32) to systemic invasion that restrict population diversity and limit the potential for transposon insertion site analysis of infection in neonatal rats with GI colonization.

## RESULTS

### Generation of a Tn*5* mutant library and identification of essential genes.

**FIG 1:**
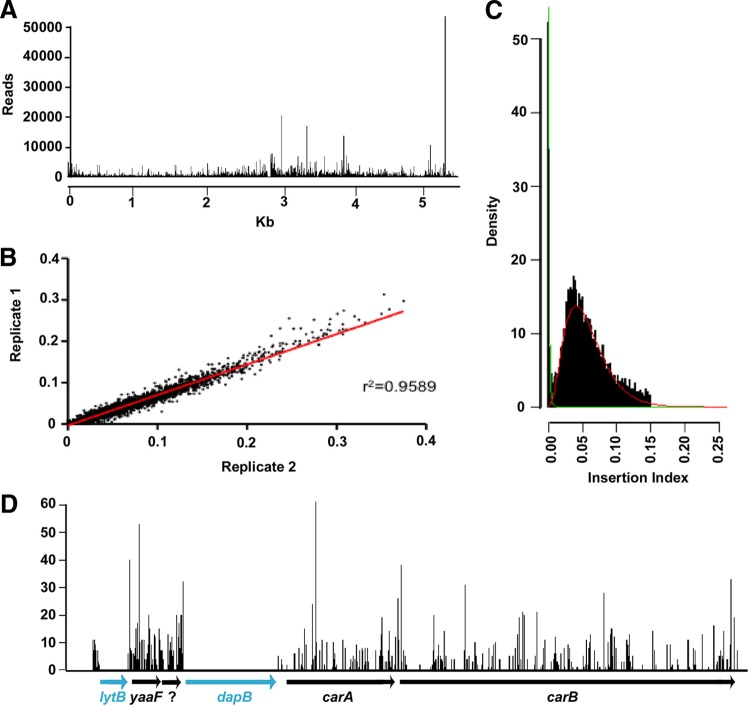
High-density transposon library for identification of genes essential for *in vitro* growth of E. coli K1 A192PP. (A) Distribution of Tn*5* insertions along the E. coli K1 A192PP genome. The number of sequence reads mapped to each single genomic location is plotted to show a representation of the entire genome. (B) Insertion index values for two biological replicates are strongly correlated. (C) Density plot showing the frequency of insertion index values for all genes. A bimodal distribution is evident, with the left peak representing “essential” genes in which a Tn*5* insertion is lethal for growth on selective Luria-Bertani agar; the right peak represents “nonessential” genes into which Tn*5* inserted without an induction of lethality. Green and red lines indicate gamma distributions used to estimate likelihood ratios and *P* values. (D) Tn*5* insertion site reads plotted to a 9-kb region of the E. coli A192PP genome. The height of each line on the *y* axis indicates the number of reads at each Tn*5* insertion site. The genes *lytB* and *dapB* possess no insertion sites, indicating that they are putative essential genes.

To provide sufficient saturation density for the identification of E. coli K1 genes essential for growth *in vitro* and of those conferring fitness in a range of defined environments, approximately 300 individual pools, each with 1 × 10^3^ to 5 × 10^3^ transposon mutants of E. coli A192PP, were constructed and combined to form a library containing over 7.75 × 10^5^ mutants. Linker PCR was performed on randomly selected mutants to confirm that Tn*5* had inserted into random genomic locations (see Fig. S1 in the supplemental material). TraDIS was performed on pooled but uncultured mutants to identify Tn*5* insertion sites within the 5.52-Mbp genome of A192PP (33). Sequences of indexed amplicons were determined, and 2 × 10^6^ sequence reads containing Tn*5* were mapped onto the E. coli K1 A192PP genome. Reads mapped to 237,860 unique Tn*5* insertion sites and were distributed along the entire genome (Fig. 1A).

As the Tn*5* library contained a high transposon insertion density, genes with no or limited numbers of Tn*5* insertion sites are likely to be essential for growth in nutrient-replete media such as Luria-Bertani (LB) broth. We calculated insertion indices for each gene by normalizing the number of insertions in each gene by the gene length. Insertion index values for two technical replicates were highly correlated (Spearman's rho = 0.9589) (Fig. 1B). A density plot of insertion indices produced a bimodal distribution, with a narrow peak representing genes with no or a limited number of Tn*5* insertions and a broad peak containing genes with a large number of Tn*5* insertions (Fig. 1C); the former comprised genes that confer lethality when mutated, and the latter comprised genes that can be mutated without affecting bacterial viability. To identify genes that significantly lack Tn*5* insertions and therefore are essential for *in vitro* growth, gamma distributions from the density plot were used to determine log_2_ likelihood ratios. Examples of essential genes containing no or limited numbers of Tn*5* insertions are shown in Fig. 1D. A total of 357 genes were predicted to be essential for the *in vitro* growth of E. coli K1 A192PP, and these genes are shown in Table S1 in the supplemental material, together with KEGG (Kyoto Encyclopedia of Genes and Genomes) descriptors for genes involved in metabolic pathways.

**FIG 2:**
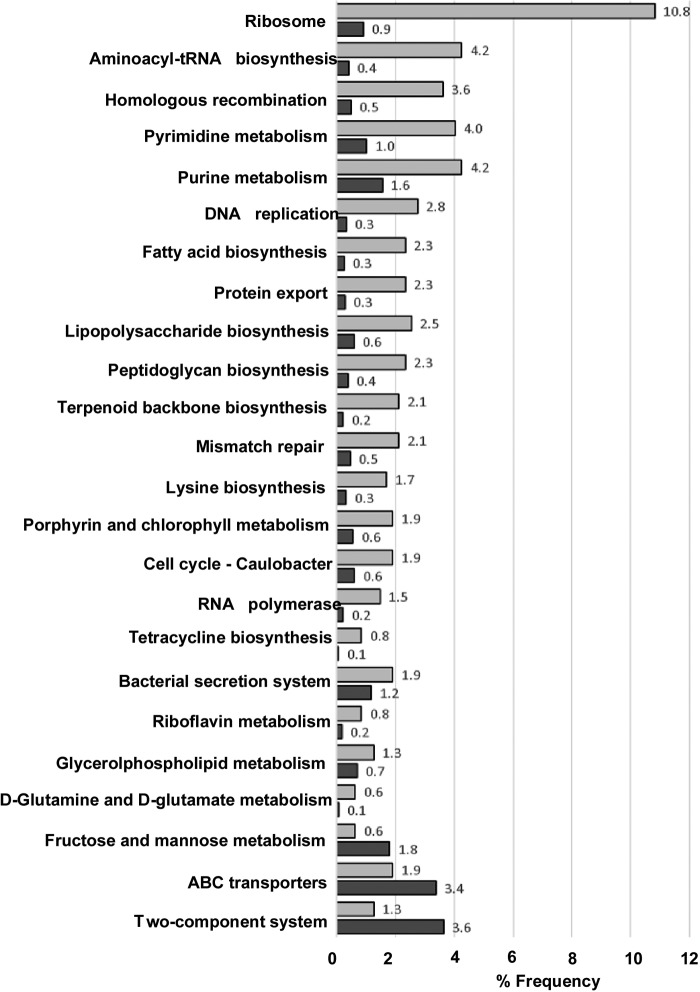
Essential E. coli A192PP genes in each selected KEGG functional ortholog (KO) category. Gene frequencies (light gray) (expressed as a percentage of essential coding DNA sequences [CDSs] for each category) are compared to their frequency within the whole genome (dark gray). KO = *x*/*y*, where *y* is the number of CDSs in the whole genome and *x* is the number of identified essential genes.

COG (Clusters of Orthologous Groups) was used to identify the functional category of each gene essential for growth *in vitro* from the A192PP whole-genome sequence (BioProject accession number PRJEB9141). Genes involved in the ribosomal structure (11% of the total number of essential genes) and protein biosynthesis (15%) featured prominently and were significantly enriched in relation to their representation within the whole genome, as were genes encoding proteins for DNA replication (3%), cell wall (peptidoglycan and lipopolysaccharide) biosynthesis (6.25%), and membrane biogenesis (3%) (Fig. 2). Genes for protein secretion and export as well as ABC transporter genes were also well represented; the remaining essential genes were involved in a wide variety of cellular catabolic and anabolic functions. The list features 254 genes that were found by TraDIS (34) to be essential for the growth of an E. coli sequence type 131 (ST131) multidrug-resistant urinary tract isolate (from a total of 315 essential genes) in Luria broth. In a similar fashion, 253 genes determined to be essential for the growth of E. coli K-12 MG1655 in LB broth were also identified as being essential in the present study (Table S1); the K-12 study employed a comprehensive set of precisely defined, in-frame, single-gene deletion mutants (35) and not transposon insertion sequencing.

### Maintaining Tn*5* library diversity.

**FIG 3:**
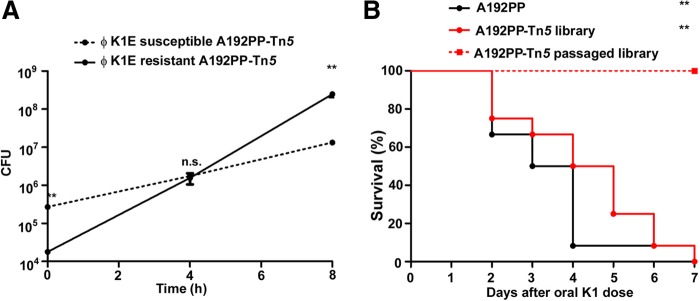
Culture of the E. coli K1 A192PP-Tn*5* library results in loss of population diversity and enrichment of nonencapsulated mutants. (A) Changes in the proportions of encapsulated and noncapsulated E. coli A192PP bacteria during culture of the E. coli A192PP-Tn*5* library in LB medium at 37°C (200 orbits/min) (*n* = 3) (±1 standard deviation) (*P* < 0.01 by Student's *t* test). CFU of encapsulated and nonencapsulated bacteria were determined from the proportion of bacteria susceptible to the K1E bacteriophage. (B) Survival of P2 rats colonized with E. coli K1 A192PP, the uncultured E. coli A192PP-Tn*5* library, and the cultured (LB broth for 8 h at 37°C) E. coli A192PP-Tn*5* library. Pups (*n* = 12 for each group) were colonized with 2 × 10^6^ to 4 × 10^6^ CFU by the oral route. ns, nonsignificant; *, *P* < 0.05; **, *P* < 0.01 (as determined by a log rank [Mantel-Cox] test to compare the survival of the cultured library with those of the wild-type strain and the uncultured library).

The polySia capsule is a major determinant of virulence in E. coli K1 and is central to the capacity of K1 clones to cause neonatal systemic infection (11, 36). PolySia biosynthesis imposes a substantial metabolic burden on producer strains (37). As TraDIS and other transposon insertion sequencing procedures generally employ growth in liquid medium for recovery and expansion of the output pool (38), we investigated the impact of batch culture on the expression of the K1 capsule within the Tn*5* library. The complete Tn*5* library was inoculated into LB broth and incubated for 8 h at 37°C, and the proportions of encapsulated and nonencapsulated A192PP bacteria were determined by susceptibility to the E. coli K1-specific bacteriophage K1E within the population. Nonencapsulated mutants initially comprised 4.66% of the bacterial population, but by the end of the incubation period, this value had risen to 98.24% (Fig. 3A). Growth rates in LB broth of E. coli A192PP and a nonencapsulated mutant of A192PP randomly selected from the Tn*5* library did not differ significantly (Fig. S2A).

The cultured Tn*5* library was avirulent, as determined by administration to P2 neonatal rat pups, whereas GI colonization with 2 × 10^6^ to 6 × 10^6^ CFU E. coli A192PP and the uncultured Tn*5* library was lethal. A similar colonizing inoculum of the cultured (8 h at 37°C) E. coli A192PP-Tn*5* library had no impact on survival, and all pups remained healthy over the 7-day observation period (Fig. 3B), even though all animals remained heavily colonized with K1 bacteria throughout the experiment (data not shown). Thus, culture of the library prior to challenge resulted in the loss of phenotypic diversity and virulence. The complete Tn*5* library contained 2.81 × 10^5^ unique Tn*5* insertions, of which 750 (2.66% of the bacterial population) possessed transposon insertions in genes determining capsule biosynthesis (data not shown). The probability that cultured sublibraries of more than 5 × 10^3^ mutants contained a noncapsulated mutant was calculated to be ≥0.98 but was only 0.55 for sublibraries of 1 × 10^3^ mutants. Low-complexity libraries of 10^3^ mutants maintained virulence in P2 neonatal rat pups after culture, whereas more-complex libraries did not (Fig. S3), due to the absence of mutants lacking the capacity to express the polySia capsule within the inoculum. To minimize bias, in all subsequent experiments, libraries of sufficient complexity to contain multiple numbers of nonencapsulated mutants were used; for experiments utilizing neonatal rats, the period between colonization initiation and tissue harvesting was kept to a minimum, and tissue homogenates were cultured directly on selective agar plates with no intervening liquid culture step.

### Genes required for GI colonization.

E. coli A192PP bacteria colonize the small intestine of neonatal rats following oral administration of the bacterial bolus, with 10^7^ to 10^8^ K1 bacteria/g intestinal tissue persisting for at least 1 week (12, 13). Translocation of the neonatal pathogen to the blood compartment via the mesenteric lymphatic system occurs predominantly, and in all likelihood exclusively, across the epithelium of the midsection of the small intestine (MSI), even though the density of colonizing bacteria in this region of the GI tract is no higher than that within neighboring proximal small intestine (PSI) or distal small intestine (DSI) locations (12).

**FIG 4:**
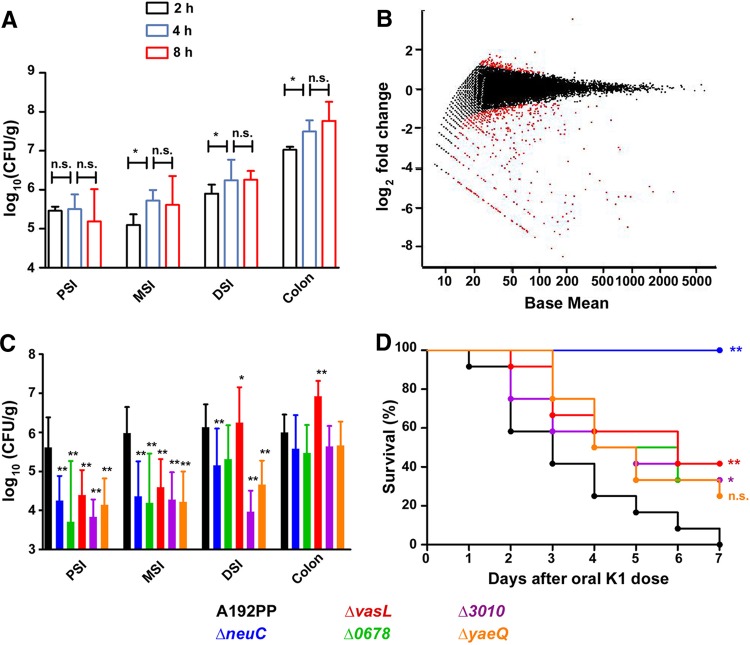
Identification of genes promoting GI colonization of E. coli A192PP in neonatal rats by using a high-density transposon library. (A) Colonization of PSI, MSI, DSI, and colon after oral administration of 2 × 10^6^ to 6 × 10^6^ CFU E. coli K1 A192PP to P2 pups. (B) Log_2_ fold change and average Tn*5* insertion site read abundance for each gene after MSI colonization of P2 rat pups (*n* = 4) over a 4-h stabilization period, expressed as an MA plot. An inoculum containing 2 × 10^4^ unique E. coli K1 A192PP-Tn*5* mutants was prepared, and 1 × 10^9^ CFU were administered orally. E. coli colonies (2 × 10^5^) were recovered from the inoculum (input pool) and from MSI homogenates (output pool) by culture on LB agar containing 50 μg/ml kanamycin. Red data points represent Tn*5* insertion sites determined as being differentially expressed in the output pool compared to the input pool by using a negative binomial test with a false discovery rate of 0.1. (C) Colonization of P2 rat intestine by E. coli K1 A192PP and single-gene mutants. Bacteria (2 × 10^6^ to 4 × 10^6^ CFU) were administered orally to P2 rats (*n* = 12/group). Pups were sacrificed, and the E. coli K1 burden in intestinal sections was enumerated 24 h after the initiation of colonization. Parent and mutant strain CFU values were compared by using Student's *t* test (*, *P* < 0.05; **, *P* < 0.01). (D) Survival of P2 rats colonized with E. coli K1 A192PP and single-gene mutants. Bacteria (2 × 10^6^ to 4 × 10^6^ CFU) were administered orally to P2 rats (*n* = 12/group). ns, nonsignificant; *, *P* < 0.05; **, *P* < 0.01 (as determined by a log rank [Mantel-Cox] test).

Few attempts have been made to determine the genes or gene products required by E. coli K1 for colonization of the GI tract (39). To prevent a loss of diversity of the E. coli K1 A192PP-Tn*5* library, we minimized the period of colonization before sampling the E. coli K1 population of the MSI. The colonizing E. coli K1 population in proximal, middle, and distal regions of the small intestine did not expand beyond 4 h after the initiation of colonization (Fig. 4A); GI tissues were therefore excised at this time point. To identify mutants with a decreased capacity to colonize the MSI, P2 rats were fed 1 × 10^9^ CFU of an E. coli K1 A192PP-Tn*5* library containing 2 × 10^5^ mutants, the pups were sacrificed after 4 h, and E. coli K1 bacteria in the MSI were enumerated. The bacterial load in rats colonized with the Tn*5* library was comparable to that in rats colonized with the wild-type strain (data not shown). MSI tissues from four rats were pooled, homogenized, and cultured on LB agar containing kanamycin to ensure that the mutant frequency was not overestimated by the inclusion of measurements of DNA from dead bacteria; Kan^r^ colonies were then pooled, DNA was extracted, and the fitness of each mutant was determined by TraDIS. Input and output pools each comprised 2 × 10^5^ CFU, and the ratios of input to MSI read counts were expressed as log_2_ fold changes. A wide distribution of fitness scores (40) was detected (Fig. 4B). The majority of transposon insertions did not have a strong negative or positive effect on colonization of the MSI. A total of 387 transposon insertions, within 167 genes, showed significantly decreased normalized read counts between input and output pools (negative log_2_ fold change and *P* < 0.05) (see Table S2 in the supplemental material). Of the 387 insertion sites, 180 were not detectable in the output pool, demonstrating a complete loss in the output pool. Many of these transposon insertion sites occurred within the same gene (Table S2). For example, within the *neuC* gene, 70 unique transposon insertion sites were identified as being lost during colonization. Transposon-interrupted genes were identified as being important for colonization of the MSI and were grouped into seven arbitrary categories: (i) genes encoding surface structures, including pili; (ii) genes encoding secretory components; (iii) genes involved in intermediary metabolism; (iv) stress response genes; (v) cytoplasmic membrane (CM)-located genes; (vi) genes for iron acquisition; and (vii) others and hypothetical genes.

High proportions of mutations associated with a decreased MSI-colonizing capacity were located in genes affecting the biosynthesis of surface structures (Table S2). A few genes were involved in lipopolysaccharide (LPS) biosynthesis (*yrbH* and *yiaH*) and outer membrane (OM) protein biosynthesis (*ompG* and *ycbS*), but the majority affected the polySia capsule, with genes of the *neu* operon (41) accounting for 194 of the 387 colonization-attenuated mutants. There is some evidence that capsular polysaccharides may promote adhesion to biological and nonbiological surfaces during biofilm formation (37), but there has been little or no consideration of a role for capsules as mediators of GI colonization.

A limited number of genes associated with type II and IV secretion were identified as being required for colonization of the MSI; these multiprotein complexes translocate a wide range of proteins and protein complexes across host membranes (42, 43) and are implicated in adherence and intestinal colonization by enterohemorrhagic E. coli in farm animals (44). Genes for the assembly of pilus proteins, including some carried on the *tra* locus, which are likely to be located on plasmids that initiate conjugation, were also linked to colonization; pili are virulence factors that may mediate attachment to and infection of host cells (45). Colonization by both commensals and pathogens is dependent on nutrient scavenging, sensing of chemical signals, and regulation of gene expression as the bacteria adapt to a new and potentially hostile environment that in the case of E. coli K1 appears to rely on stress response genes such as *yhiM* (which encodes a protein aiding survival at low pH), the heat shock protein genes *clpB* and *yrfH*, as well as DNA repair genes. A large number of genes encoded enzymes involved in the metabolism of sugars (e.g., *gcd*, *rpiR*, and *glgC*), amino acids (*dadX*, *metB*, and *tdcB*), fatty acids (*yafH* and *fixA*), growth factors (*bisC*, *yigB*, and *thiF*), and other secondary metabolites (*yicP*). Transporters and permeases involved in central intermediary metabolism were also featured prominently: these proteins included permeases of the major facilitator superfamily (YjiZ), the hexose phosphate transport protein UhpT, the carnitine transporter CaiT, and a range of CM-located sugar transporters. Of note was the impact of a mutation of the *fucR* l-fucose operon activator on colonization; fucose is abundant in the GI tract, and the fucose-sensing system in enterohemorrhagic E. coli regulates colonization and controls the expression of virulence and metabolic genes (46). The availability of free iron is severely limited in the GI tract, and ingestion of iron predisposes an individual to infection (47); the importance of iron acquisition for E. coli K1 during GI colonization is reflected in the requirement for a number of genes related to iron uptake (e.g., *feoB* and *fepA*).

### GI tract-colonizing capacity and virulence of single-gene mutants.

To investigate the contribution of the polySia capsule to the colonization of the neonatal rat GI tract, we disrupted the *neuC* gene of E. coli A192PP using bacteriophage λ Red recombinase to produce a capsule-free mutant as judged by resistance to E. coli K1-specific phage K1E. We also produced other single-gene mutants for genes identified by the TraDIS GI screen: *vasL* (encoding a type IV secretion system protein), *yfeC* (predicted to form part of a toxin/antitoxin locus), and two genes with unknown function, *yaeQ* and A192PP_3010 (the latter is present in genomes of other extraintestinal E. coli pathogens, including IHE3034, UTI89, RS218, PMV-1, and S88). Growth rates of these mutants, in particular the capsule-negative *neuC* mutant (see Fig. S2B in the supplemental material), were indistinguishable from that of the E. coli A192PP parent in LB medium. All mutants were examined for their capacity to colonize the GI tract and cause lethal infection in P2 rat pups (Fig. 4C and D).

The E. coli A192PP parent strain or single-gene mutants (2 × 10^6^ to 6 × 10^6^ CFU) were administered orally to P2 rats; all members of a litter of 12 pups received the same strain. Pups were sacrificed 24 h after initiation of colonization, and E. coli K1 bacteria in the small intestine (PSI, MSI, and DSI) and colon were enumerated. The capacity of all mutants to transit through the upper portion of the alimentary canal, pass through the stomach, and colonize the small intestine was markedly inferior to that of the wild-type strain (Fig. 4C). Reductions in colonization of the PSI, MSI, and DSI by the mutants, including E. coli A192PPΔ*neuC*::*kan*, were significant, with the only exception being colonization of the DSI by A192PPΔ*yfeC*::*kan*, with no significant difference between the parent and mutant. Interestingly, no increases in the numbers of viable A192PPΔ*neuC*::*kan*, A192PPΔ*vasL*::*kan*, A192PPΔ*3010*::*kan*, and A192PPΔ*yaeQ*::*kan* bacteria recovered from the colon were noted to compensate for reductions in the colonization of the small intestine. There was a significant increase in the colonic burden of viable A192PPΔ*yfeC*::*kan* bacteria compared to that of bacteria of the parent strain. We established previously (12) that E. coli A192PP transits to the blood circulation via the mesenteric lymphatic system by exploiting a vesicular pathway through the GI epithelium only at the MSI. As numbers of mutant bacteria colonizing this region of the small intestine were much reduced compared to those of the parent strain, we determined the capacity of the single-gene mutants to elicit lethal systemic infection following GI colonization by oral administration of 2 × 10^6^ to 6 × 10^6^ bacteria at P2 (Fig. 4D). Four of the five mutants (A192PPΔ*neuC*::*kan*, A192PPΔ*vasL*::*kan*, A192PPΔ*3010*::*kan*, and A192PPΔ*yfeC*::*kan*) displayed significantly reduced lethal potential compared to that of the A192PP parent. The loss of capsule (A192PPΔ*neuC*::*kan*) resulted in a complete loss of lethality over the 7-day observation period. The administration of A192PPΔ*vasL*::*kan* elicited a lethal response in 41.6% of pups; 33.3% and 25% of pups survived after receiving A192PPΔ*3010*::*kan* and A192PPΔ*yfeC*::*kan*, respectively, at P2. For A192PPΔ*yaeQ*::*kan*, 75% of pups succumbed to lethal infection, but this did not reach levels of significance compared to the 100% lethality engendered by the A192PP parent (*P* > 0.05). Overall, these data indicate that the TraDIS screen efficiently identified genes important for MSI colonization that impact pathogenic potential.

### A bottleneck to infection in the neonatal rat prevents identification of genes for translocation across the gastrointestinal epithelium.

Our initial intention was to exploit the high degree of susceptibility of P2 neonatal rats to systemic infection, sepsis, and meningitis following oral administration of an effective dose of E. coli A192PP bacteria in order to determine all genes required to enable the neonatal pathogen to overcome previously defined (12–15) physical and immunological barriers to invasion of the blood circulation and dissemination to the meninges. However, previous studies indicated that relatively few E. coli K1 bacteria migrate from colonized sites within the GI tract to the blood (10), constraining the genetic diversity of the translocated bacterial population and eliminating genotypes from the translocated gene pool in a stochastic manner that does not reflect the fitness of individual genes to contribute to genotypes with invasive potential (32). We therefore determined if there were bottlenecks that would compromise the identification of mutants with an attenuated capacity to translocate from the GI tract to the blood compartment; if any experimental bottlenecks are narrower than the complexity of the E. coli A192PP Tn*5* library, many relevant transposon insertion mutants will be lost entirely by chance (38). Furthermore, the existence of a restrictive bottleneck would limit the complexity of the library that could be used for TraDIS evaluation of populations colonizing the MSI (input pool) and reaching the blood (output pool).

**FIG 5:**
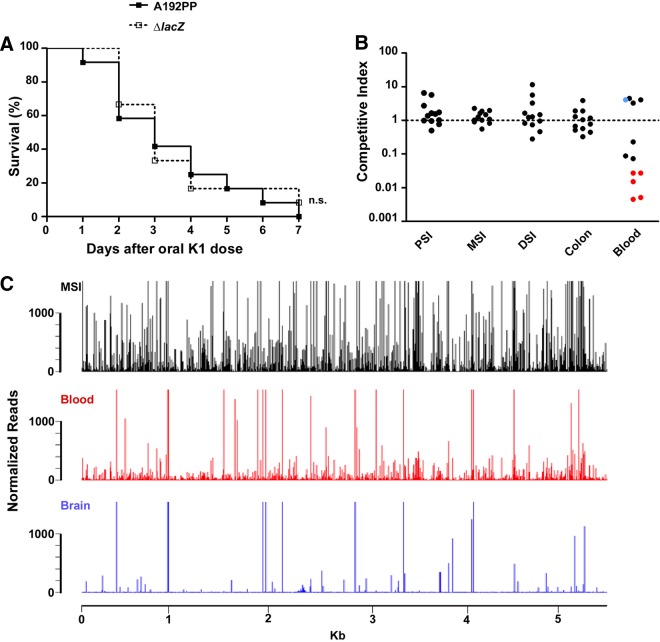
Bottleneck to systemic infection in the neonatal rat. (A) Survival of rats colonized at P2 by oral administration of E. coli K1 A192PP or A192PPΔ*lacZ*::*kan* (*n* = 12 pups for both groups). ns, nonsignificant; *, *P* < 0.05; **, *P* < 0.01 (as determined by a log-rank [Mantel-Cox] test). (B) Competitive indices of intestinal colonization and gut-to-blood transit of E. coli K1 A192PP and A192PPΔ*lacZ*::*kan*. A 1:1 mixture of E. coli K1 A192PP and A192PPΔ*lacZ*::*kan* (total of 2 × 10^6^ to 4 × 10^6^ CFU) was administered orally to P2 pups. After 24 h, ratios of A192PP and A192PPΔ*lacZ*::*kan* bacteria were enumerated in segmented GI tissues and in the blood, as indicated, using selective media. Data for animals in which only E. coli K1 A192PP or A192PPΔ*lacZ*::*kan* was detected in the blood are shown in red and blue, respectively, indicating the existence of a bottleneck to infection. (C) Loss of diversity of E. coli K1 A192PP-Tn*5* populations recovered from the blood (red) and brain (blue) following translocation from the GI tract (black).

We constructed the E. coli A192PPΔ*lacZ* mutant by bacteriophage λ Red recombineering and confirmed that there was no significant difference in lethal potential between E. coli A192PP and the *lacZ* mutant (Fig. 5A). We then used mixtures of parent and mutant bacteria to investigate the existence of bottlenecks that restrict translocation to the blood compartment. A 1:1 mixture (total of 2 × 10^6^ to 4 × 10^6^ CFU) of E. coli A192PP and A192PPΔ*lacZ* was administered orally to P2 rat pups, the animals were sacrificed after 24 h, and GI tissue homogenates were plated for the quantification of bacteria of each strain. The competitive index (CI), the ratio of input A192PP and A192PPΔ*lacZ* bacteria to output A192PP and A192PPΔ*lacZ* bacteria, was calculated for excised PSI, MSI, DSI, colon, and mesenteric lymphatic tissues and for blood. CI values for the PSI, MSI, DSI and colon were not significantly different from 1 (one-sample *t* test), indicating that the composition of the colonizing inoculum was maintained in each rat pup (Fig. 5B). However, there was more heterogeneity in CI values of bacterial populations from the blood, and in five pups, only one strain could be recovered from the blood (the parent strain only for four animals and A192PPΔ*lacZ* only for one animal). The highly restrictive bottleneck between GI epithelial transport and entry into the blood circulation supports the argument that the reduced virulence of the complete, cultured library in comparison to that of less-complex sublibraries (Fig. S3) is due at least in part to a reduced likelihood that a fully virulent mutant would randomly escape capture by the mesenteric lymphatic system. The presence of significant bottlenecks between the GI tract, blood circulation, and brain was confirmed by determination of the complexity of recovered Tn*5* library populations from these sources (Fig. 5C).

### Identification of E. coli K1 A192PP genes required for survival in human serum.

Systemic infection in neonatal rats is likely to be maintained only if E. coli A192PP bacteria survive in the blood circulation. Due to the limited exposure to antigens *in utero* coupled with deficits in adaptive immunity, neonates depend on innate immunity for protection against infection. The complement system provides frontline innate defense against Gram-negative bacterial infection, and the polySia capsule in turn enables E. coli K1 to avoid successful complement-mediated attack by host immune mechanisms. To obtain insights into E. coli K1 pathogenesis during the invasive phase of infection, and in light of restrictions placed on the neonatal rat model with regard to the use of TraDIS by the gut-to-blood bottleneck, we used the E. coli A192PP Tn*5* library to investigate genes essential for A192PP fitness in pooled normal human serum, a reliable and plentiful source of all soluble components of the three complement pathways (48).

**FIG 6:**
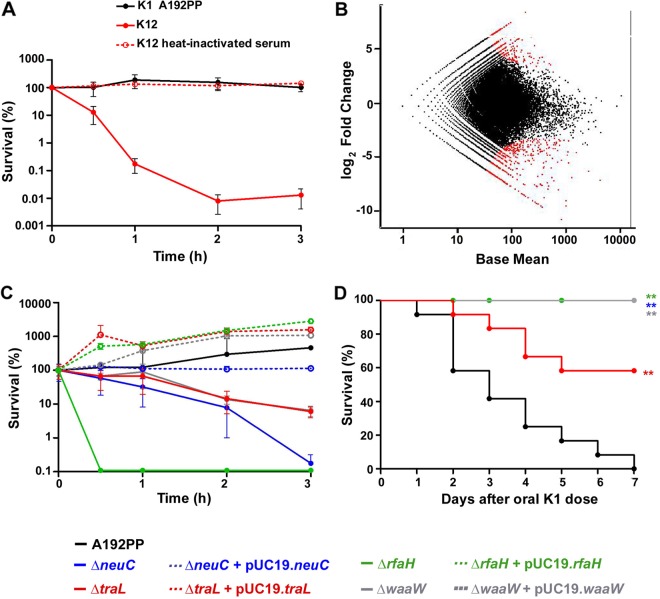
Identification of genes contributing to the complement resistance of E. coli A192PP by using a high-density transposon library. (A) Survival of E. coli A192PP and E. coli K-12 strain MG1655 in 22% pooled human serum. The latter was used a serum-susceptible control (*n* = 3); error bars represent ranges of values. (B) Log_2_ fold change and average Tn*5* insertion site read abundance for each gene after incubation of 1 × 10^6^ CFU containing 2 × 10^4^ unique E. coli K1 A192PP-Tn*5* mutants in 22% pooled human serum for 3 h at 37°C. Colonies (2 × 10^5^) were obtained by culture of diluted aliquots on LB agar containing 50 μg/ml kanamycin. The inoculum served as the input pool. Red data points represent Tn*5* insertion sites determined as being differentially expressed in the output pool compared to the input pool using a negative binomial test with a false discovery rate of 0.1. (C) Survival of 1 × 10^6^ E. coli K1 A192PP bacteria and single-gene mutants in 22% normal human and heat-inactivated (56°C for 30 min) serum. The final volume of the reaction mixture was 1.5 ml (*n* = 3); error bars represent ranges of values. Complementation with the functional gene restored resistance in all cases. (D) Survival of P2 rats colonized with E. coli K1 A192PP and single-gene mutants. Bacteria (2 × 10^6^ to 4 × 10^6^) were administered orally to P2 rats (*n* = 12/group). ns, nonsignificant; *, *P* < 0.05; **, *P* < 0.01 (as determined by a log rank [Mantel-Cox] test).

E. coli A192PP is resistant to the bactericidal action of human serum (Fig. 6A). A portion of the A192PP-Tn*5* library containing 2 × 10^4^ mutants (1 × 10^9^ CFU) was incubated in either 30% human serum or 30% heat-inactivated serum (final volume of 375 μl) at 37°C for 3 h. Kan^r^ bacteria in the input and output pools (each with 2 × 10^5^ bacteria) were collected, DNA was extracted from each pool, and transposon insertion sites were sequenced. A wide distribution of fitness scores was detected (Fig. 6B). Mutation of 97 genes (negative log_2_ fold change and *P* < 0.05) resulted in decreased survival in normal serum but not in heat-inactivated serum (Fig. 6C; see also Table S3 in the supplemental material).

A high proportion of genes identified in the TraDIS screen as contributing to resistance encoded cell surface constituents. It is well established that the polySia capsule protects E. coli K1 from complement attack (16, 17), and three mutations in the *kps* capsule gene cluster compromised serum survival. The central region of the cluster contains the *neu* genes that direct the biosynthesis, activation, and polymerization of the *N*-acetylneuraminic acid building block of polySia. *neuC* encodes the UDP *N*-acetylglucosamine 2-epimerase that catalyzes the formation of *N*-acetylmannosamine (49), and the *O*-acetyltransferase *neuD* acetylates monomeric neuraminic acid at carbon position 7 or 9 (50). KpsM is a component of the multimeric ATP-binding cassette transporter involved in the translocation of the polySia capsule through a transmembrane corridor to the cell surface (41, 51). A disruption of the genes encoding these proteins will prevent polySia expression (41); the interruption of *rfaH*, identified in the TraDIS screen, will also prevent capsule expression, but its loss will have a more profound effect on the surface topography of E. coli A192PP, as this transcriptional antiterminator is required for the expression of operons that direct the synthesis, assembly, and export of LPS core components, pili, and toxins in addition to the capsule (52, 53). Indeed, survival in serum is dependent on antitermination control by RfaH (54). Another identified gene that impacts capsule formation was *bipA*; BipA is a tyrosine-phosphorylated GTPase that regulates a variety of cell processes, including some associated with virulence, through the ribosome (55, 56). Other genes involved in LPS biosynthesis and pilus formation were also identified: *waaW* encodes a UDP-galactose:(galactosyl) LPS alpha1,2-galactosyltransferase involved in the synthesis of the R1 and R4 LPS core oligosaccharides (57), and *wzzE* encodes a polysaccharide copolymerase that catalyzes the polymerization of LPS O-antigen oligosaccharide repeat units into a mature polymer within the periplasmic space in readiness for export to the cell surface (58). Both mutations will prevent the attachment of LPS O-antigen side chains to the core oligosaccharide of LPS. The 16 genes that specify pilus synthesis that were identified in the screen included the majority of the genes of the *tra* locus.

The TraDIS screen identified a range of proteins that are embedded in the OM (Fig. 6C), none of which had been previously implicated in complement resistance. These proteins are likely to influence the topography of the bacterial surface. Of the remaining genes with an assigned function, the majority were involved in cell metabolism and the stress response; it is well established that metabolic processes are intimately associated with the process of complement-mediated killing of bacteria (59, 60). To verify the screen, we constructed four single-gene mutants of E. coli A192PP by bacteriophage λ Red recombineering. Genes with roles in LPS synthesis (*rfaH* and *waaW*), capsule synthesis (*neuC*), and pilus assembly (*traL*) were mutated; none of these mutants showed any reduction in growth rates in LB broth. All mutants displayed significant reductions in complement resistance following incubation in pooled human serum (Fig. 6D). E. coli A192PPΔ*rfaH* was exquisitely susceptible, with no colonies being detected after 30 min. The viability of A192PPΔ*neuC* was also compromised, with a log reduction in viability over the 3-h incubation period of 3-fold. Killing of A192PPΔ*traL* and A192PPΔ*waaW* was less marked, but these mutations significantly reduced viability. Complementation of the mutants with the functional gene introduced on a pUC19 vector completely restored resistance in all cases (Fig. 6D). These genes also contributed to lethality in P2 neonatal rats (Fig. 6E). The lethal capacity of A192PPΔ*neuC*, A192PPΔ*rfaH*, and A192PPΔ*waaW* was completely attenuated in comparison to that of E. coli A192PP; 42% of pups administered A192PPΔ*traL* succumbed to systemic infection (all *P* < 0.01).

## DISCUSSION

Systemic infection with meningeal involvement arises spontaneously after GI colonization of neonatal rats with a high proportion of E. coli K1 isolates, and the pathway to infection mirrors to a large extent that of natural infections in the human host. In contrast to models of bacterial infection that create artificial pathogenesis by bypassing some or all of the barriers to infection by the injection of a bacterial bolus directly into the blood circulation, the neonatal rat model provides an opportunity to investigate the progress of the pathogen as it transits from gut to blood to brain in a stepwise fashion. TraDIS and other transposon sequencing methods enable the simultaneous and rapid determination of the fitness contribution of every gene under a given condition and therefore have the potential to enable the identification of genes that are essential for, or significantly contribute to, each step of the infection process. However, stochastic loss will become evident if each mutant in the input pool does not have an equal chance to overcome the physical, physiological, and immunological barriers presented by the host (61). This was clearly the case with the epithelial transit of E. coli A192PP, with evidence that on occasion systemic infection arose due to only one viable bacterial cell entering the blood circulation (Fig. 5B), and complements other studies showing single-cell or low-cell-number bottlenecks in models of severe infection (62–64). As translocation from colonization sites within the MSI to the blood was not amenable to analysis by TraDIS, we determined genes essential for survival in the presence of complement, a major component of the innate immune system that protects against extracellular systemic pathogens (17).

The high density of transposon insertions into random genomic positions along the entire E. coli A192PP chromosome, with minimal insertional bias (Fig. 1A), enabled the identification of genes essential for growth in nutrient-replete LB medium. Of the 357 E. coli A192PP genes considered essential, orthologues of 254 (from 315) were previously identified using TraDIS in a multidrug-resistant uropathogenic strain of E. coli ST131 grown in LB broth (34), and 253 were identified in an E. coli K-12 strain (35), confirming the existence of a core set of essential genes in E. coli. As anticipated, a high proportion of these genes encoded enzymes involved in a range of key metabolic functions such as carbohydrate, protein, and nucleobase metabolism, and the remainder were associated with essential functions such as transport, cell organization, and biogenesis.

During characterization of the E. coli A192PP mutant library, we examined the impact of culture in liquid medium on the expression of the polySia capsule, which places large demands on cell energy expenditure, as lengthy incubation times before marker selection may decrease library complexity (38). Unexpectedly, we found that prolonged culture of the library enriched the proportion of nonencapsulated mutants (Fig. 3A). We anticipated that the loss of capsule would enable the nonencapsulated mutants to grow at a higher rate than those of capsule-replete mutants and the wild type and to outcompete capsule-bearing library members. However, the growth of a nonencapsulated mutant selected at random from the library was virtually identical to, and not significantly different from, that of the E. coli A192PP parent strain (see Fig. S2A in the supplemental material). There was also no difference in the climax populations of the strains at the end of the logarithmic phase of growth. In a similar fashion, the growth curve for a *neuC* single-gene mutant was identical to that of E. coli A192PP (Fig. S2B). *neuC* is involved in the synthesis of the *N*-acetylneuraminic acid monomeric unit of polySia and, as a consequence, is unable to elaborate the capsule. It is clearly impractical to evaluate the growth kinetics of every distinct nonencapsulated mutant in the Tn*5* library, but it currently appears that differences in growth rates of individual library members cannot explain the highly reproducible enrichment that we observed. Indeed, the use of transposon insertion libraries is predicated on the assumption that there are no significant differences in the growth rates of individual mutants. At present, the basis of the loss of mutants expressing capsule in TraDIS library cultures cannot be readily explained.

A sublibrary of 2 × 10^5^ mutants was used to establish genes involved in GI colonization. To minimize bias due to any outgrowth of nonencapsulated mutants on the GI epithelium, we harvested E. coli K1 from the MSI after 4 h, by which time maximal CFU had been achieved; bacteria were plated directly on solid medium to further avoid outgrowth. Bias due to this restricted timeline is likely to be low, as the majority of genes involved in adhesion and complement resistance are expressed constitutively. TraDIS identified the polySia capsule as a major determinant of GI colonization associated with E. coli K1. There is little or no evidence from the literature that capsules of Gram-negative bacteria enhance GI colonization; indeed, it has been reported that they interfere with adhesive interactions by obstructing the binding of underlying surface molecules to mucosal surfaces (65, 66). The E. coli A192PPΔ*neuC*::*kan* single-gene mutant displayed a reduced capacity to colonize the MSI (Fig. 4C), although it should be borne in mind that passage through the upper alimentary canal and stomach may impact the number of mutant bacteria gaining access to the small intestine. In this context, it should be noted that capsular exopolysaccharide protects E. coli from the environmental stress of stomach acid (67).

Other cell surface structures that are likely to have an impact on adhesion to and colonization of the mucosal layer associated with the MSI were identified by TraDIS. Pili are established mediators of adhesion of E. coli to the host epithelium, although a large proportion of the evidence comes from enterotoxigenic and enteropathogenic strains (68, 69). LPS and OM protein-encoding genes were also implicated, as were genes involved in the stress response, reflecting ongoing adaptation to a new and hostile environment. The involvement of genes encoding metabolic enzymes, including some for anaerobic respiration, equates to increases in bacterial cell numbers in the anaerobic environment of the small intestine, and for iron acquisition genes, this reflects the low availability of intestinal luminal iron (47, 70). Genes encoding some components of type II and type IV secretion systems were found at decreased frequencies in the output pool. Members of these gene categories were also identified previously by Martindale et al. (39) as being necessary for GI colonization by E. coli K1 fecal isolate RS228 using signature-tagged mutagenesis; no genes found in that study were identified in the present study, in spite of the close genetic relatedness of the strains employed.

The intestinal lumen represents a potentially important portal of entry for pathogens into the host through adhesion, invasion, or disruption of the epithelial barrier (71). In neonatal rats, E. coli K1 induces no detectable disruption of barrier integrity but exploits an intracellular pathway to access the submucosa (12). Only small numbers of bacteria breach the mesenteric lymphatic barrier in an apparently random fashion (Fig. 5), and this precludes analysis by TraDIS. To accumulate data on genes and gene products facilitating invasion and survival/replication in the blood circulation, we examined essentiality for avoiding complement-mediated bactericidal effects. Although not all E. coli K1 isolates from cases of systemic infection are resistant to complement, resistance is encountered more frequently among K1 and K5 capsular types than among other K types (72); E. coli O18:K1 strains (such as A192) are in turn resistant more often than are other O:K serotype combinations (73) due to the capacity of the polySia capsule to prevent complement activation. It is assumed, but not established, that the polySia capsule surrounding susceptible strains does not completely mask either OM-located activators of complement or lipid domains on the outer surface of the cell that are targets for OM intercalation of the C5b-9 membrane attack complex, the entity responsible for bacterial killing (59). In addition, long and numerous LPS O-antigen side chains are necessary but not sufficient to enable the target cell to avoid complement killing (74), and they are able to bind the C1 inhibitor to arrest classical or lectin pathway activation at the early C1 stage (75). The importance of these structures for the complement resistance of E. coli K1 is supported by the decreased frequency of key LPS and capsule genes in the output pool along with a large number of OM-embedded proteins.

A small number of OM proteins, such as TraT and Iss, have been implicated as being determinants of complement resistance (74), but they have been introduced into low-resistance backgrounds in high copy numbers; their role in the intrinsic resistance of clinical isolates is unclear, and no mechanisms have been invoked to account for increases in resistance. The insertion of large numbers of protein molecules into the OM may fortuitously alter the biophysical properties of the bilayer, reducing the surface area and fluidity of lipid patches that are essential for the binding and assembly of the C5b-9 membrane attack complex. The identification of a range of OM proteins as being putative complement resistance determinants by TraDIS creates an opportunity to systematically investigate their precise function through the generation of single-gene mutants, and we intend to pursue this line of investigation. We suggest that the architecture of the external surface of the OM, together with other more-external macromolecular structures such as polysaccharide capsules, influences the capacity of the pore-generating C5b-9 complex to perturb the integrity of the OM. Thus, the surface of susceptible strains contains a sufficient number of exposed lipid domains to facilitate C5b-9 generation and penetration, whereas the spatial and temporal organization of the OM of resistant bacteria is dominated by supramolecular protein assemblages to a degree where insufficient hydrophobic domains are available to act as C5b-9 assembly and binding sites, and this state persists throughout the growth cycle. The data that we have generated in this study are compatible with this hypothesis. An array of metabolic genes emerged as being essential for the maintenance of the complement-resistant phenotype (Fig. 6D) and may be indicative of repair processes invoked due to complement attack. The exposure of resistant E. coli to complement results in a minor perturbation of membrane integrity and metabolic homeostasis (76, 77), and C5b-9 intercalation into the OM has profound effects on cellular metabolic parameters (60).

TraDIS was also employed by Phan and coworkers to define the serum resistome of a globally disseminated, multidrug-resistant clone of E. coli ST131 (34). Those researchers identified, and in most cases validated, 56 genes that contributed to the high level of complement resistance displayed by this pathogen. In a fashion similar to that in our study, genes involved in the synthesis and expression of cell surface components were prominent. A number of genes contributing to LPS biosynthesis, such as those of the *waa* operon, the *wzz* locus, and *rfaH*, were common to both studies, as was the gene encoding the intermembrane protein AcrA. Genes of the plasmid-borne *tra* locus, which we determined to be components of the E. coli A192PP serum resistome, were not present in E. coli ST131 (34), but other OM-located proteins may fulfill a similar role in reducing the fluidic properties of the bilayer. In contrast to the well-established role of the E. coli K1 polysialyl polymer in the prevention of complement activation, no capsule genes were identified as components of the serum resistome of E. coli ST131, but different ST131 isolates express different capsule types due to extensive mosaicism at the capsule locus (78), and these uronic acid-containing polymers are unlikely to prevent complement activation (75). Thus, the different strategies employed by the two strains to prevent successful complement attack, together with differences in bacterial surface composition and topography, probably explain variations in the serum resistomes of these related pathogens.

In summary, we identified E. coli K1 genes required for growth in standard laboratory liquid medium and for colonization of the GI tract of P2 neonatal rat pups. Both data sets provide insights into the biology of K1 neuropathogens and could provide the basis for drug discovery programs for the identification of selective antibacterial or colonization-inhibiting agents. In our rodent model, the stochastic nature of invasion of the blood and probably brain prevented TraDIS analysis of gene essentiality for crossing gut epithelial and choroid plexus borders, but some indication of genes necessary for survival in blood was obtained from analyses of output pools after incubation of E. coli A192PP in human serum, a potent source of complement.

## MATERIALS AND METHODS

### Ethics statement.

Animal experiments were approved by the Ethical Committee of the University College London (UCL) School of Pharmacy and the United Kingdom Home Office and were conducted in accordance with national legislation.

### Bacteria and culture conditions.

E. coli strain A192PP was obtained by serial passage of E. coli A192 (serotype O18:K1), isolated from a patient with septicemia (79), in P2 neonatal rats as described previously (11). Carriage of the polysialyl K1 capsule was determined with phage K1E (80): colonies were streaked onto Mueller-Hinton (MH) agar, 10 μl of a phage suspension containing ∼10^9^ PFU/ml was dropped onto each streak, the plates were incubated overnight at 37°C, and the proportion of encapsulated bacteria within cultures was quantified by comparing the ratio of phage-susceptible to phage-resistant colonies. E. coli A192PP single-gene mutants (Table 1) were constructed by using bacteriophage λ Red recombination (81); the oligonucleotides employed for the construction of targeted mutants, for the confirmation of targeted mutants, and for the construction of complemented mutants are shown in Tables S3 to S5 in the supplemental material. All bacteria were cultured in LB medium and on LB agar at 37°C; media were supplemented with either 100 μg/ml ampicillin or 50 μg/ml kanamycin as required.

**TABLE 1:**
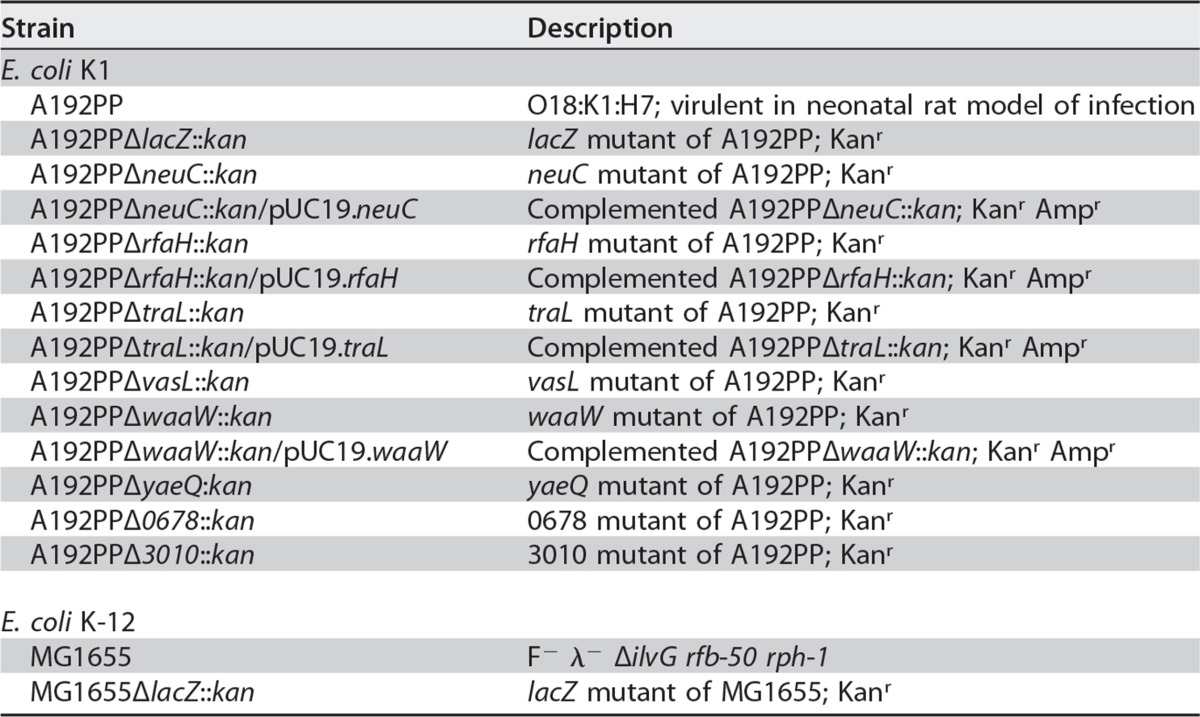
Strains used in this study

### Tn*5* library construction.

The EZ-Tn*5* ⟨KAN-2⟩ Tnp transposome (Epicentre Biotechnologies) was introduced into E. coli A192PP by electroporation. Transformants were selected by growth on LB plates containing 50 μg/ml kanamycin overnight. Pools of 1 × 10^3^ to 5 × 10^3^ colonies were collected and frozen at −80°C in phosphate-buffered saline (PBS) containing 20% glycerol. Aliquots of individual pools were combined to create larger populations of mutants of up to 7.75 × 10^5^ mutants. Genomic DNA was extracted from 1-ml cultures by using the PurElute bacterial genomic kit (Edge Biosystems) according to standard protocols.

### Linker PCR of Tn*5* insertion sites.

Linker PCR was used to test individual transformant colonies and to confirm individual random-insertion events. DNA (2.5 μg) was digested with the AluI restriction enzyme (Promega) and purified by using a MinElute PCR purification kit (Qiagen). A linker, formed by annealing of oligonucleotides 254 (5′-CGACTGGACCTGGA-3′) and 256 (5′-GATAAGCAGGGATCGGAACCTCCAGGTCCAGTCG-3′), was ligated to purified fragments (50 ng) with a Quick ligation kit (NEB). Linker PCR was performed with linker- and transposon-specific oligonucleotides (258 [5′-GATAAGCAGGGATCGGAACC-3′] and 5′-GCAATGTAACATCAGAGATTTTGAG-3′, respectively) by using a HotStart *Taq* Mastermix kit (Qiagen) and thermocycling conditions of 95°C for 5 min; 35 cycles of 94°C for 45 s, 56°C for 1 min, and 72°C for 1 min; and 72°C for 10 min. The resulting amplicons were separated on 1.5% agarose gels at 100 V for 60 min.

### Illumina sequencing.

For sequencing of Tn*5* insertion sites, approximately 2 μg of genomic DNA was degraded to ∼500-bp fragments by ultrasonication using a Covaris instrument. Fragments were end repaired and A tailed by using the NEBNext DNA library preparation reagent kit for Illumina sequencing (NEB). The adapters Ind_Ad_T (ACACTCTTTCCCTACACGACGCTCTTCCGATC*T, where * indicates phosphorothionate) and Ind_Ad_B (GATCGGAAGAGCGGTTCAGCAGGAATGCCGAGACCGATCTC) were annealed and ligated to DNA fragments. PCR was performed with the transposon- and adapter-specific primers Tn-FO (5′-TCGTCGGCAGCGTCAGATGTGTATAAGAGACAGCGGGGATCCTCTAGAGTCGACCTGC-3′) and Adapt-RO (5′-GTCTCGTGGGCTCGGAGATGTGTATAAGAGACAGACACTCTTTCCCTACACGACGCTCTTCCGATC-3′). Tn-FO and Adapt-RO contain a forward overhang and a reverse overhang for indexing of amplicons by Nextera index primers (Illumina). PCR was performed by using a HotStart *Taq* Mastermix kit (Qiagen) and thermocycling conditions of 95°C for 5 min; 22 cycles of 94°C for 45 s, 56°C for 1 min, and 72°C for 1 min; and 72°C for 10 min. The resulting amplicons were separated on 1.5% agarose gels at 70 V for 90 min, and those between 150 and 700 bp were selected and purified by using a QIAquick gel extraction kit (Qiagen). Samples were indexed with oligonucleotides from the Nextera XT index kit (Illumina) by using HotStart ReadyMix (Kapa Biosystems) and with thermocycling conditions of 95°C for 3 min; 8 cycles of 95°C for 30 s, 55°C for 30 s, and 72°C for 30 s; and 72°C for 5 min applied. Indexed amplicons were purified by using the AMPure XP system (Agencourt). The final concentration of samples was confirmed by using Qubit dsDNA BR assays (ThermoFisher Scientific). Indexed amplicons were sequenced on an Illumina Mi-Seq platform as 151-bp paired-end reads according to the manufacturer's protocol (Illumina).

### Bioinformatic and statistical analyses.

Raw sequence reads that passed Trimmomatic quality control filters (82) and contained the Tn*5* transposon were mapped to the E. coli K1 A192PP reference genome (14) by using Bowtie (83), permitting zero mismatches and excluding reads that did not map to a single site. The reference genome assembly contains open reading frames (ORFs) located on contigs that were mapped to the IHE3034 chromosome and ORFs located on other contigs that are likely to map to plasmids and other mobile genetic elements. An in-house pipeline based on the SAMtools (http://samtools.sourceforge.net/) and BCFtools toolkits was utilized for the alignment files to determine insertion sites and coverage. To identify essential and nonessential genes, the insertion index was calculated for each gene by dividing the number of unique insertions in the gene by the gene length. Observed insertion index values were fitted to a bimodal distribution with a gamma distribution (or an exponential distribution for genes with no observed insertion sites) corresponding to essential and nonessential genes. The log_2_ likelihood, and corresponding *P* values, of each gene belonging to essential or nonessential sets was calculated by using R software. To compare the fitnesses of individual mutants in input and output populations, reads were normalized and tested for differential base means by calculating log_2_ fold changes and corresponding *P* values at a false discovery rate of 0.1 using DESeq with R software.

### Colonization and infection of neonatal rats.

Timed-birth Wistar rat pup litters (usually *n* = 12) were purchased from Harlan UK, delivered at P2, and colonized on the same day. Pups were retained throughout each experiment with the natural mothers in a single dedicated cage under optimal conditions (19°C to 21°C, 45 to 55% humidity, 15 to 20 changes of air/h, and a 12-h light/dark cycle) and were returned to the mother immediately after colonization. Mothers had unrestricted access to standard rat chow and water. The procedure was described in detail previously (84). In brief, all members of P2 rat pup litters were fed 20 μl of mid-logarithmic-phase E. coli bacteria (2 × 10^6^ to 6 × 10^6^ CFU unless otherwise stated) from an Eppendorf micropipette. GI colonization was confirmed by culture of perianal swabs on MacConkey agar, and bacteremia was detected by MacConkey agar culture of blood taken postmortem. Disease progression was monitored by daily evaluation of symptoms of systemic infection, and neonates were culled by decapitation and recorded as dead once a threshold had been reached: pups were regularly examined for skin color, agility, agitation after abdominal pressure, the presence of a milk line, temperature, weight, and behavior in relation to the mother. Neonates were culled immediately when abnormalities for three of these criteria were evident. After sacrifice, GI tissues were excised aseptically without washing, the colon was separated, and the SI was segmented into 2-cm portions representing proximal, middle, and distal small intestinal tissues. Tissues were then transferred to ice-cold phosphate-buffered saline and homogenized. Bacteria were quantified by serial dilution culture on MacConkey agar supplemented with 25 μg/ml kanamycin as appropriate. The presence of E. coli K1 was confirmed with phage K1E: 20 lactose-fermenting colonies were streaked onto MH agar, 10 μl of a phage suspension containing ∼10^9^ PFU/ml was dropped onto each streak, and the plates were incubated overnight. E. coli K1 bacteria were quantified by multiplying the total CFU by the proportion of K1E-susceptible colonies. In all cases, at least 19 colonies were susceptible to the K1 phage; E. coli K1 was never found in samples from noncolonized pups.

### Susceptibility to human serum.

Serum was obtained from healthy volunteers and used immediately. Bacteria were grown to late logarithmic phase in LB broth in an orbital incubator (minimum of 200 orbits/min), and 500 μl of the culture was removed, washed twice with gelatin-Veronal-buffered saline plus magnesium and calcium ions (pH 7.35) (GVB^++^), and suspended in an equal volume of GVB^++^. Fresh human serum was diluted 1:3 in GVB^++^ and prewarmed to 37°C. Bacterial suspensions and serum solutions were mixed 1:2 to give a final concentration of ∼10^7^ CFU/ml and incubated at 37°C for 3 h in a total volume of 125 μl containing 22% serum. Surviving E. coli bacteria were quantified by serial dilution and incubation on LB agar overnight. Prewarmed, heat-inactivated (56°C for 30 min) serum served as a control.

### Accession number(s).

Raw read data for all transposon insertions have been deposited in the European Nucleotide Archive (ENA). All files are located at https://www.ebi.ac.uk/ena/data/view/PRJEB24291; accession numbers are as follows: ERR2235345 and ERR2235346 for the identification of essential genes for replicates 1 and 2, ERR2235567 for the input population, ERR2235568 for the output population of rat MSI genes, ERR2235569 for the output population of serum-exposed E. coli A192PP, and ERR2235570 for the output population of bacteria exposed to heat-inactivated serum.

## Supplementary Material

Supplemental material

## References

[1] Simonsen KA, Anderson-Berry AL, Delair SF, Davies HD 2014 Early-onset neonatal sepsis. Clin Microbiol Rev 27:21–47. doi:10.1128/CMR.00031-13. 24396135 PMC3910904

[2] Bonacorsi S, Bingen E 2005 Molecular epidemiology of *Escherichia coli* causing neonatal meningitis. Int J Med Microbiol 295:373–381. doi:10.1016/j.ijmm.2005.07.011. 16238014

[3] Tsai MH, Lee CW, Chu SM, Lee IT, Lien R, Huang HR, Chiang MC, Fu RH, Hsu JF, Huang YC 2016 Infectious complications and morbidities after neonatal bloodstream infections: an observational cohort study. Medicine (Baltimore) 95:e3078. doi:10.1097/MD.0000000000003078. 26986139 PMC4839920

[4] Robbins JB, McCracken GH, Gotschlich EC, Ørskov F, Ørskov I, Hanson LA 1974 *Escherichia coli* K1 capsular polysaccharide associated with neonatal meningitis. N Engl J Med 290:1216–1220. doi:10.1056/NEJM197405302902202. 4133095

[5] Korhonen TK, Valtonen MV, Parkkinen J, Väisänen-Rhen V, Finne J, Ørskov F, Ørskov I, Svenson SB, Mäkelä PH 1985 Serotypes, hemolysin production, and receptor recognition of *Escherichia coli* strains associated with neonatal sepsis and meningitis. Infect Immun 48:486–491. 2580792 10.1128/iai.48.2.486-491.1985 PMC261353

[6] Rutishauser U 2008 Polysialic acid in the plasticity of the developing and adult vertebrate nervous system. Nat Rev Neurosci 9:26–35. doi:10.1038/nrn2285. 18059411

[7] Sarff LD, McCracken GH, Schiffer MS, Glode MP, Robbins JB, Ørskov I, Ørskov F 1975 Epidemiology of *Escherichia coli* K1 in healthy and diseased newborns. Lancet i:1099–1104. 10.1016/s0140-6736(75)92496-4 49468

[8] Tunkel AR, Scheld WM 1993 Pathogenesis and pathophysiology of bacterial meningitis. Clin Microbiol Rev 6:118–136. doi:10.1128/CMR.6.2.118. 8472245 PMC358273

[9] Glode MP, Sutton A, Moxon ER, Robbins JB 1977 Pathogenesis of neonatal *Escherichia coli* meningitis: induction of bacteremia and meningitis in infant rats fed *E. coli* K1. Infect Immun 16:75–80. 326679 10.1128/iai.16.1.75-80.1977 PMC421490

[10] Pluschke G, Mercer A, Kusećek B, Pohl A, Achtman M 1983 Induction of bacteremia in newborn rats by *Escherichia coli* K1 is correlated with only certain O (lipopolysaccharide) antigen types. Infect Immun 39:599–608. 6187683 10.1128/iai.39.2.599-608.1983 PMC347994

[11] Mushtaq N, Redpath MB, Luzio JP, Taylor PW 2004 Prevention and cure of systemic *Escherichia coli* K1 infection by modification of the bacterial phenotype. Antimicrob Agents Chemother 48:1503–1508. doi:10.1128/AAC.48.5.1503-1508.2004. 15105097 PMC400570

[12] Birchenough GMH, Dalgakiran F, Witcomb LA, Johansson MEV, McCarthy AJ, Hansson GC, Taylor PW 2017 Postnatal development of the small intestinal mucosa drives age-dependent regio-selective susceptibility to *Escherichia coli* K1 infection. Sci Rep 7:83. doi:10.1038/s41598-017-00123-w. 28250440 PMC5427930

[13] Birchenough GMH, Johansson MEV, Stabler RA, Dalgakiran F, Hansson GC, Wren BW, Luzio JP, Taylor PW 2013 Altered innate defenses in the neonatal gastrointestinal tract in response to colonization by neuropathogenic *Escherichia coli*. Infect Immun 81:3264–3275. doi:10.1128/IAI.00268-13. 23798529 PMC3754193

[14] Witcomb LA, Collins JW, McCarthy AJ, Frankel G, Taylor PW 2015 Bioluminescent imaging reveals novel patterns of colonization and invasion in systemic *Escherichia coli* K1 experimental infection in the neonatal rat. Infect Immun 83:4528–4540. doi:10.1128/IAI.00953-15. 26351276 PMC4645386

[15] Zelmer A, Bowen M, Jokilammi A, Finne J, Luzio JP, Taylor PW 2008 Differential expression of the polysialyl capsule during blood-to-brain transit of neuropathogenic *Escherichia coli* K1. Microbiology 154:2522–2532. doi:10.1099/mic.0.2008/017988-0. 18667585 PMC2572004

[16] Meri S, Pangburn MK 1990 Discrimination between activators and nonactivators of the alternative pathway of complement: regulation via a sialic acid/polyanion binding site on factor H. Proc Natl Acad Sci U S A 87:3982–3986. doi:10.1073/pnas.87.10.3982. 1692629 PMC54028

[17] Taylor PW 1993 Non-immunoglobulin activators of the complement system, p 37–68. *In* Sim RB (ed), Activators and inhibitors of complement. Kluwer Academic Publishers, Dordrecht, The Netherlands.

[18] Nassif X, Bourdoulous S, Eugène E, Couraud PE 2002 How do extracellular pathogens cross the blood-brain barrier? Trends Microbiol 10:227–232. doi:10.1016/S0966-842X(02)02349-1. 11973156

[19] Brouwer MC, Tunkel AR, van de Beek D 2010 Epidemiology, diagnosis, and antimicrobial treatment of acute bacterial meningitis. Clin Microbiol Rev 23:467–492. doi:10.1128/CMR.00070-09. 20610819 PMC2901656

[20] Johnson JR, Oswald E, O'Bryan TT, Kuskowski MA, Spanjaard L 2002 Phylogenetic distribution of virulence-associated genes among *Escherichia coli* isolates associated with neonatal bacterial meningitis in the Netherlands. J Infect Dis 185:774–784. doi:10.1086/339343. 11920295

[21] Payros D, Secher T, Boury M, Brehin C, Ménard S, Salvador-Cartier C, Cuevas-Ramos G, Watrin C, Marcq I, Nougayrède JP, Dubois D, Bedu A, Garnier F, Clermont O, Denamur E, Plaisancié P, Theodorou V, Fioramonti J, Olier M, Oswald E 2014 Maternally acquired genotoxic *Escherichia coli* alters offspring's intestinal homeostasis. Gut Microbes 5:313–325. doi:10.4161/gmic.28932. 24971581 PMC4153768

[22] McCarthy AJ, Martin P, Cloup E, Stabler RA, Oswald E, Taylor PW 2015 The genotoxin colibactin is a determinant of virulence in *Escherichia coli* K1 experimental neonatal systemic infection. Infect Immun 83:3704–3711. doi:10.1128/IAI.00716-15. 26150540 PMC4534652

[23] Garcie C, Tronnet S, Garénaux A, McCarthy AJ, Brachmann AO, Pénary M, Houle S, Nougayrède JP, Piel J, Taylor PW, Dozois CM, Genevaux P, Oswald E, Martin P 2016 The bacterial stress-responsive Hsp90 chaperone is required for the production of the genotoxin colibactin and the siderophore yersiniabactin by *Escherichia coli*. J Infect Dis 214:916–924. doi:10.1093/infdis/jiw294. 27412582

[24] Langridge GC, Phan MD, Turner DJ, Perkins TT, Parts L, Haase J, Charles I, Maskell DJ, Peters SE, Dougan G, Wain J, Parkhill J, Turner AK 2009 Simultaneous assay of every *Salmonella* Typhi gene using one million transposon mutants. Genome Res 19:2308–2316. doi:10.1101/gr.097097.109. 19826075 PMC2792183

[25] van Opijnen T, Bodi KL, Camilli A 2009 Tn-seq: high-throughput parallel sequencing for fitness and genetic interaction studies in microorganisms. Nat Methods 6:767–772. doi:10.1038/nmeth.1377. 19767758 PMC2957483

[26] Goh KGK, Phan MD, Forde BM, Chong TM, Yin WF, Chan KG, Ulett GC, Sweet MJ, Beatson SA, Schembri MA 2017 Genome-wide discovery of genes required for capsule production by uropathogenic *Escherichia coli*. mBio 8:e01558–17. doi:10.1128/mBio.01558-17. 29066548 PMC5654933

[27] van Opijnen T, Camilli A 2013 Transposon insertion sequencing: a new tool for systems-level analysis of microorganisms. Nat Rev Microbiol 11:435–442. doi:10.1038/nrmicro3033. 23712350 PMC3842022

[28] Bachman MA, Breen P, Deornellas V, Mu Q, Zhao L, Wu W, Cavalcoli JD, Mobley HL 2015 Genome-wide identification of Klebsiella pneumoniae fitness genes during lung infection. mBio 6:e00775-15. doi:10.1128/mBio.00775-15. 26060277 PMC4462621

[29] Wang N, Ozer EA, Mandel MJ, Hauser AR 2014 Genome-wide identification of Acinetobacter baumannii genes necessary for persistence in the lung. mBio 5:e01163–14. doi:10.1128/mBio.01163-14. 24895306 PMC4049102

[30] Skurnik D, Roux D, Aschard H, Cattoir V, Yoder-Himes D, Lory S, Pier GB 2013 A comprehensive analysis of *in vitro* and *in vivo* genetic fitness of *Pseudomonas aeruginosa* using high-throughput sequencing of transposon libraries. PLoS Pathog 9:e1003582. doi:10.1371/journal.ppat.1003582. 24039572 PMC3764216

[31] Subashchandrabose S, Smith SN, Spurbeck RR, Kole MM, Mobley HL 2013 Genome-wide detection of fitness genes in uropathogenic *Escherichia coli* during systemic infection. PLoS Pathog 9:e1003788. doi:10.1371/journal.ppat.1003788. 24339777 PMC3855560

[32] Abel S, Abel zur Wiesch P, Davis BM, Waldor MK 2015 Analysis of bottlenecks in experimental models of infection. PLoS Pathog 11:e1004823. doi:10.1371/journal.ppat.1004823. 26066486 PMC4465827

[33] McCarthy AJ, Negus D, Martin P, Pechincha C, Oswald E, Stabler RA, Taylor PW 2016 Pathoadaptive mutations of *Escherichia coli* K1 in experimental neonatal systemic infection. PLoS One 11:e0166793. doi:10.1371/journal.pone.0166793. 27861552 PMC5115809

[34] Phan MD, Peters KM, Sarkar S, Lukowski SW, Allsopp LP, Gomes Moriel D, Achard ME, Totsika M, Marshall VM, Upton M, Beatson SA, Schembri MA 2013 The serum resistome of a globally disseminated multidrug resistant uropathogenic *Escherichia coli* clone. PLoS Genet 9:e1003834. doi:10.1371/journal.pgen.1003834. 24098145 PMC3789825

[35] Baba T, Ara T, Hasegawa M, Takai Y, Okumura Y, Baba M, Datsenko KA, Tomita M, Wanner BL, Mori H 2006 Construction of *Escherichia coli* K-12 in-frame, single-gene knockout mutants: the Keio collection. Mol Syst Biol 2:2006.0008. doi:10.1038/msb4100050. PMC1681482 16738554

[36] Gonzalez MD, Lichtensteiger CA, Vimr ER 2001 Adaptation of signature-tagged mutagenesis to *Escherichia coli* K1 and the infant-rat model of invasive disease. FEMS Microbiol Lett 198:125–128. doi:10.1111/j.1574-6968.2001.tb10630.x. 11430402

[37] Roberts IS 1996 The biochemistry and genetics of capsular polysaccharide production in bacteria. Annu Rev Microbiol 50:285–315. doi:10.1146/annurev.micro.50.1.285. 8905082

[38] Chao MC, Abel S, Davis BM, Waldor MK 2016 The design and analysis of transposon insertion sequencing experiments. Nat Rev Microbiol 14:119–128. doi:10.1038/nrmicro.2015.7. 26775926 PMC5099075

[39] Martindale J, Stroud D, Moxon ER, Tang CM 2000 Genetic analysis of *Escherichia coli* K1 gastrointestinal colonization. Mol Microbiol 37:1293–1305. doi:10.1046/j.1365-2958.2000.02088.x. 10998163

[40] Anders S, Huber W 2010 Differential expression analysis for sequence count data. Genome Biol 11:R106. doi:10.1186/gb-2010-11-10-r106. 20979621 PMC3218662

[41] Vimr ER, Steenbergen SM 2006 Mobile contingency locus controlling *Escherichia coli* K1 polysialic acid capsule acetylation. Mol Microbiol 60:828–837. doi:10.1111/j.1365-2958.2006.05158.x. 16677296

[42] Fronzes R, Christie PJ, Waksman G 2009 The structural biology of type IV secretion systems. Nat Rev Microbiol 7:703–714. doi:10.1038/nrmicro2218. 19756009 PMC3869563

[43] Patrick M, Gray MD, Sandkvist M, Johnson TL 19 10 2010, posting date Type II secretion in *Escherichia coli*. EcoSal Plus 2010 doi:10.1128/ecosalplus.4.3.4. 26443782

[44] Ho TD, Davis BM, Ritchie JM, Waldor MK 2008 Type 2 secretion promotes enterohemorrhagic *Escherichia coli* adherence and intestinal colonization. Infect Immun 76:1858–1865. doi:10.1128/IAI.01688-07. 18316380 PMC2346697

[45] Kline KA, Dodson KW, Caparon MG, Hultgren SJ 2010 A tale of two pili: assembly and function of pili in bacteria. Trends Microbiol 18:224–232. doi:10.1016/j.tim.2010.03.002. 20378353 PMC3674877

[46] Pacheco AR, Curtis MM, Ritchie JM, Munera D, Waldor MK, Moreira CG, Sperandio V 2012 Fucose sensing regulates bacterial intestinal colonization. Nature 492:113–117. doi:10.1038/nature11623. 23160491 PMC3518558

[47] Weinberg ED 2009 Iron availability and infection. Biochim Biophys Acta 1790:600–605. doi:10.1016/j.bbagen.2008.07.002. 18675317

[48] Serruto D, Rappuoli R, Scarselli M, Gros P, van Strijp JA 2010 Molecular mechanisms of complement evasion: learning from staphylococci and meningococci. Nat Rev Microbiol 8:393–399. doi:10.1038/nrmicro2366. 20467445

[49] Vann WF, Daines DA, Murkin AS, Tanner ME, Chaffin DO, Rubens CE, Vionnet J, Silver RP 2004 The NeuC protein of *Escherichia coli* K1 is a UDP *N*-acetylglucosamine 2-epimerase. J Bacteriol 186:706–712. doi:10.1128/JB.186.3.706-712.2004. 14729696 PMC321479

[50] Steenbergen SM, Lee YC, Vann WF, Vionnet J, Wright LF, Vimr ER 2006 Separate pathways for O acetylation of polymeric and monomeric sialic acids and identification of sialyl *O*-acetyl esterase in *Escherichia coli* K1. J Bacteriol 188:6195–6206. doi:10.1128/JB.00466-06. 16923886 PMC1595355

[51] Pigeon RP, Silver RP 1997 Analysis of the G93E mutant allele of KpsM, the membrane component of an ABC transporter involved in polysialic acid translocation in *Escherichia coli* K1. FEMS Microbiol Lett 156:217–222. doi:10.1111/j.1574-6968.1997.tb12730.x. 9513268

[52] Bailey MJ, Hughes C, Koronakis V 1997 RfaH and the *ops* element, components of a novel system controlling bacterial transcription elongation. Mol Microbiol 26:845–851. doi:10.1046/j.1365-2958.1997.6432014.x. 9426123

[53] Hu K, Artsimovitch I 2017 A screen for rfaH suppressors reveals a key role for a connector region of termination factor Rho. mBio 8:e00753–17. doi:10.1128/mBio.00753-17. 28559482 PMC5449661

[54] Garrett SB, Garrison-Schilling KL, Cooke JT, Pettis GS 2016 Capsular polysaccharide production and serum survival of *Vibrio vulnificus* are dependent on antitermination control by RfaH. FEBS Lett 590:4564–4572. doi:10.1002/1873-3468.12490. 27859050

[55] Rowe S, Hodson N, Griffiths G, Roberts IS 2000 Regulation of the *Escherichia coli* K5 capsule gene cluster: evidence for the roles of H-NS, BipA, and integration host factor in regulation of group 2 capsule gene clusters in pathogenic *E. coli*. J Bacteriol 182:2741–2745. doi:10.1128/JB.182.10.2741-2745.2000. 10781541 PMC101981

[56] Krishnan K, Flower AM 2008 Suppression of Δ*bipA* phenotypes in *Escherichia coli* by abolishment of pseudouridylation at specific sites on the 23S rRNA. J Bacteriol 190:7675–7683. doi:10.1128/JB.00835-08. 18820021 PMC2583627

[57] Heinrichs DE, Yethon JA, Whitfield C 1998 Molecular basis for structural diversity in the core regions of the lipopolysaccharides of *Escherichia coli* and *Salmonella enterica*. Mol Microbiol 30:221–232. doi:10.1046/j.1365-2958.1998.01063.x. 9791168

[58] Kalynych S, Cherney M, Bostina M, Rouiller I, Cygler M 2015 Quaternary structure of WzzB and WzzE polysaccharide copolymerases. Protein Sci 24:58–69. doi:10.1002/pro.2586. 25307743 PMC4282412

[59] Taylor PW, Kroll HP 1985 Effect of lethal doses of complement on the functional integrity of target enterobacteria. Curr Top Microbiol Immunol 121:135–158. 3910366 10.1007/978-3-642-45604-6_7

[60] Dankert JR, Esser AF 1987 Bacterial killing by complement. C9-mediated killing in the absence of C5b-8. Biochem J 244:393–399. doi:10.1042/bj2440393. 3311029 PMC1148004

[61] Eckert SE, Dziva F, Chaudhuri RR, Langridge GC, Turner DJ, Pickard DJ, Maskell DJ, Thomson NR, Stevens MP 2011 Retrospective application of transposon-directed insertion site sequencing to a library of signature-tagged mini-Tn*5*Km2 mutants of *Escherichia coli* O157:H7 screened in cattle. J Bacteriol 193:1771–1776. doi:10.1128/JB.01292-10. 21278291 PMC3067669

[62] Moxon ER, Murphy PA 1978 *Haemophilus influenzae* bacteremia and meningitis resulting from survival of a single organism. Proc Natl Acad Sci U S A 75:1534–1536. 306628 10.1073/pnas.75.3.1534 PMC411507

[63] Barnes PD, Bergman MA, Mecsas J, Isberg RR 2006 *Yersinia pseudotuberculosis* disseminates directly from a replicating bacterial pool in the intestine. J Exp Med 203:1591–1601. doi:10.1084/jem.20060905. 16754724 PMC2118325

[64] Kono M, Zafar MA, Zuniga M, Roche AM, Hamaguchi S, Weiser JN 2016 Single cell bottlenecks in the pathogenesis of *Streptococcus pneumoniae*. PLoS Pathog 12:e1005887. doi:10.1371/journal.ppat.1005887. 27732665 PMC5061371

[65] Favre-Bonte S, Joly B, Forestier C 1999 Consequences of reduction of *Klebsiella pneumoniae* capsule expression on interactions of this bacterium with epithelial cells. Infect Immun 67:554–561. 9916058 10.1128/iai.67.2.554-561.1999 PMC96354

[66] Taylor CM, Roberts IS 2005 Capsular polysaccharides and their role in virulence. Contrib Microbiol 12:55–66. doi:10.1159/000081689. 15496776

[67] Mao Y, Doyle MP, Chen J 2001 Insertion mutagenesis of *wca* reduces acid and heat tolerance of enterohemorrhagic *Escherichia coli* O157:H7. J Bacteriol 183:3811–3815. doi:10.1128/JB.183.12.3811-3815.2001. 11371548 PMC95261

[68] Mu XQ, Savarino SJ, Bullitt E 2008 The three-dimensional structure of CFA/I adhesion pili: traveler's diarrhea bacteria hang on by a spring. J Mol Biol 376:614–620. doi:10.1016/j.jmb.2007.10.067. 18166195 PMC2265596

[69] Cleary J, Lai LC, Shaw RK, Straatman-Iwanowska A, Donnenberg MS, Frankel G, Knutton S 2004 Enteropathogenic *Escherichia coli* (EPEC) adhesion to intestinal epithelial cells: role of bundle-forming pili (BFP), EspA filaments and intimin. Microbiology 150:527–538. doi:10.1099/mic.0.26740-0. 14993302

[70] Cherayil BJ, Ellenbogen S, Shanmugam NN 2011 Iron and intestinal immunity. Curr Opin Gastroenterol 27:523–528. doi:10.1097/MOG.0b013e32834a4cd1. 21785351 PMC3734539

[71] Doran KS, Banerjee A, Disson O, Lecuit M 2013 Concepts and mechanisms: crossing host barriers. Cold Spring Harb Perspect Med 3:a10090. doi:10.1101/cshperspect.a010090. PMC3685877 23818514

[72] Falkenhagen U, Zingler G, Naumann G 1991 Serum resistance in different serotypes of *Escherichia coli*. Zentralbl Bakteriol 275:216–222. doi:10.1016/S0934-8840(11)80068-X. 1718305

[73] Pluschke G, Mayden J, Achtman M, Levine RP 1983 Role of the capsule and the O antigen in resistance of O18:K1 *Escherichia coli* to complement-mediated killing. Infect Immun 42:907–913. 6196296 10.1128/iai.42.3.907-913.1983 PMC264385

[74] Taylor PW 1995 Resistance of bacteria to complement, p 49–64. *In* Roth JA, Bolin CA, Brogden KA, Minion FC, Wannemuehler MJ (ed), Virulence mechanisms of bacterial pathogens, 2nd ed ASM Press, Washington, DC.

[75] Rautemaa R, Meri S 1999 Complement-resistance mechanisms of bacteria. Microbes Infect 1:785–794. doi:10.1016/S1286-4579(99)80081-1. 10816084

[76] Kroll HP, Bhakdi S, Taylor PW 1983 Membrane changes induced by exposure of *Escherichia coli* to human serum. Infect Immun 42:1055–1066. 6358036 10.1128/iai.42.3.1055-1066.1983 PMC264407

[77] Taylor PW, Kroll HP 1984 Interaction of human complement proteins with serum-sensitive and serum-resistant strains of *Escherichia coli*. Mol Immunol 21:609–620. doi:10.1016/0161-5890(84)90046-4. 6379418

[78] Alqasim A, Scheutz F, Zong Z, McNally A 2014 Comparative genome analysis identifies few traits unique to the *Escherichia coli* ST131 H30Rx clade and extensive mosaicism at the capsule locus. BMC Genomics 15:830. doi:10.1186/1471-2164-15-830. 25269819 PMC4192283

[79] Achtman M, Mercer A, Kusecek B, Pohl A, Heuzenroeder M, Aaronson W, Sutton A, Silver RP 1983 Six widespread bacterial clones among *Escherichia coli* K1 isolates. Infect Immun 39:315–335. 6218094 10.1128/iai.39.1.315-335.1983 PMC347943

[80] Gross RJ, Cheasty T, Rowe B 1977 Isolation of bacteriophages specific for the K1 polysaccharide antigen of *Escherichia coli*. J Clin Microbiol 6:548–550. 338623 10.1128/jcm.6.6.548-550.1977 PMC274821

[81] Datsenko KA, Wanner BL 2000 One-step inactivation of chromosomal genes in *Escherichia coli* K-12 using PCR products. Proc Natl Acad Sci U S A 97:6640–6645. doi:10.1073/pnas.120163297. 10829079 PMC18686

[82] Bolger AM, Lohse M, Usadel B 2014 Trimmomatic: a flexible trimmer for Illumina sequence data. Bioinformatics 30:2114–2120. doi:10.1093/bioinformatics/btu170. 24695404 PMC4103590

[83] Langmead B, Trapnell C, Pop M, Salzberg SL 2009 Ultrafast and memory-efficient alignment of short DNA sequences to the human genome. Genome Biol 10:R25. doi:10.1186/gb-2009-10-3-r25. 19261174 PMC2690996

[84] Dalgakiran F, Witcomb L, McCarthy A, Birchenough GMH, Taylor PW 2014 Non-invasive model of neuropathogenic *Escherichia coli* infection in the neonatal rat. J Vis Exp 92:e52018. doi:10.3791/52018. PMC4353393 25408299

