# Supplementary material for: Genome-Wide Identification by Transposon Insertion Sequencing of Escherichia coli K1 Genes Essential for In Vitro Growth, Gastrointestinal Colonizing Capacity, and Survival in Serum
Source: J Bacteriol. 2018 Mar 12;200(7):e00698-17. doi: 10.1128/JB.00698-17 (PMC5847654; doi:10.1128/JB.00698-17)
Supplement: Supplemental material [file supp_200_7_e00698-17__index.html]

Supplemental material 

# Genome-Wide Identification by Transposon Insertion Sequencing of Escherichia coli K1 Genes Essential for *In Vitro* Growth, Gastrointestinal Colonizing Capacity, and Survival in Serum

## Supplemental material

- Supplemental file 1 -

  Table S1 (Genes identified by TraDIS as essential for growth of *E. coli* A192PP in LB broth)

  XLSX, 67K
- Supplemental file 2 -

  Table S2 (*E. coli* K1 A192PP genes required for GI colonization)

  XLSX, 43K
- Supplemental file 3 -

  Table S3 (*E. coli* K1 A192PP genes required for survival in human serum)

  XLSX, 38K
- Supplemental file 4 -

  Legends to Tables S1 to S3; Fig. S1 (Tn*5* insertion site diversity), S2 (Growth kinetics of nonencapsulated mutants), and S3 (Virulence of high-complexity cultured *E. coli* A192PP-Tn*5* libraries in neonatal rats); and Tables S4 to S6 (Oligonucleotides for construction [S4] and confirmation [S5] of targeted mutants and construction of complemented mutants [S6])

  PDF, 768K
