# Supplementary material for: Genome-Wide Identification by Transposon Insertion Sequencing of Escherichia coli K1 Genes Essential for In Vitro Growth, Gastrointestinal Colonizing Capacity, and Survival in Serum
Source: J Bacteriol. 2018 Mar 12;200(7):e00698-17. doi: 10.1128/JB.00698-17 (PMC5847654; doi:10.1128/JB.00698-17)
Supplement: Supplemental material [file JB.00698-17_zjb999094671s4.pdf]

Genome-Wide Identification by Transposon Insertion Sequencing of *Escherichia coli* K1 Genes Essential for *in vitro* Growth, Gastrointestinal Colonizing Capacity and Survival in Serum

Alex J. McCarthy, Richard A. Stabler, Peter W. Taylor

LEGENDS FOR SUPPLEMENTAL TABLES

Table S1. Tab 1: Genes identified by TraDIS as essential for growth of *E. coli* A192PP in Luria-Bertani (LB) broth. Systematic ID, gene identifier in annotated A192PP genome (1); strand, strand location of coding DNA sequence (CDS); gene, predicted gene annotation; size, size of CDS (bp); function, predicted function; pvalue_essential, value of essentiality determined from gamma distribution; K12, essential for growth of *E. coli* K12 MG1655 (2); EC958, essential for growth of *E. coli* ST131 urinary isolate (3); KEGG_no, KEGG orthology number; KEGG_description, KEGG predicted function; ko_no, KEGG pathway number; ko_description, KEGG pathway description; EC_no, Enzyme Commission number (EC number) for enzyme classification. Tab 2: KEGG pathways enriched for, or depleted of, *E. coli* A192PP essential genes. KEGG pathway, KEGG pathway description; whole, total number of CDS in the *E. coli* A192PP genome for each category; Whole%, percentage of CDS for each category in the *E. coli* A192PP genome; Essential, number of CDS defined as essential by TraDIS; Essential%, percentage of CDS for each category; Dif%, Essential% minus whole%; %genome, ratio Essential:Whole (D:B) X 100.

Table S2. *E. coli* K1 A192PP genes required for GI colonization. GeneID, A192PP genome systematic gene number; Norm_in, normalised read depth in input pool; Norm_MSI, normalised read depth in from TraDIS library recovered from the middle section of the small intestine (MSI) 4 h after initiation of colonization; $\log_2$FoldChange, $\log_2$ (Norm_out/Norm_in); * indicates number approaching negative infinity due to division of zero reads in output pool; pval, p-value; Gene, predicted gene name; Function, manually curated gene function; PROKKA function, automated functional annotation using an *E. coli* custom library.

Table S3. *E. coli* K1 A192PP genes required for survival in human serum. GeneID, A192PP genome systematic gene number; Function, manually curated gene function; PROKKA function, automated functional annotation using an *E. coli* custom library. Log_2-fold change value and a *p* value for each mutant of each gene are provided.

References

1. McCarthy AJ, Negus D, Martin P, Pechincha C, Oswald E, Stabler RA, Taylor PW. 2016. Pathoadaptive mutations of *Escherichia coli* K1 in experimental neonatal systemic infection. PLoS One 11:e0166793.
2. Baba T, Ara T, Hasegawa M, Takai Y, Okumura Y, Baba M, Datsenko KA, Tomita M, Wanner BL, Mori H. 2006. Construction of Escherichia coli K-12 in-frame, single-gene knockout mutants: the Keio collection. Mol Syst Biol 2:2006.0008.
3. Phan MD, Peters KM, Sarkar S, Lukowski SW, Allsopp LP, Gomes Moriel D, Achard ME, Totsika M, Marshall VM, Upton M, Beatson SA, Schembri MA. 2013. The serum resistome of a globally disseminated multidrug resistant uropathogenic *Escherichia coli* clone. PLoS Genet 9:e1003834.

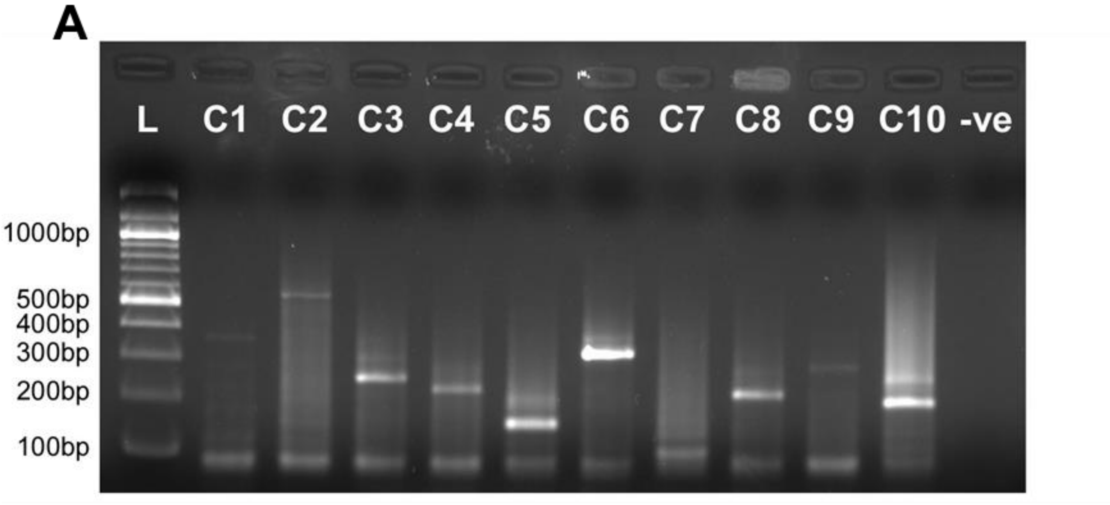

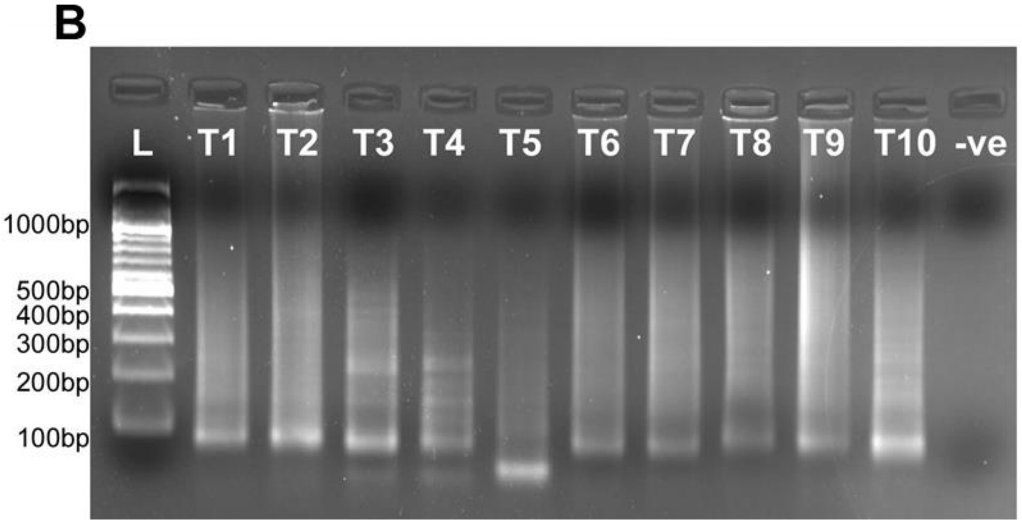

FIG S1. Linker PCR was employed to assess Tn*5* insertion site diversity in: (A) ten individual adjacent colonies grown on antibiotic-supplemented Luria-Bertani agar and (B) ten individual pools of 2000-5000 colonies each.

A

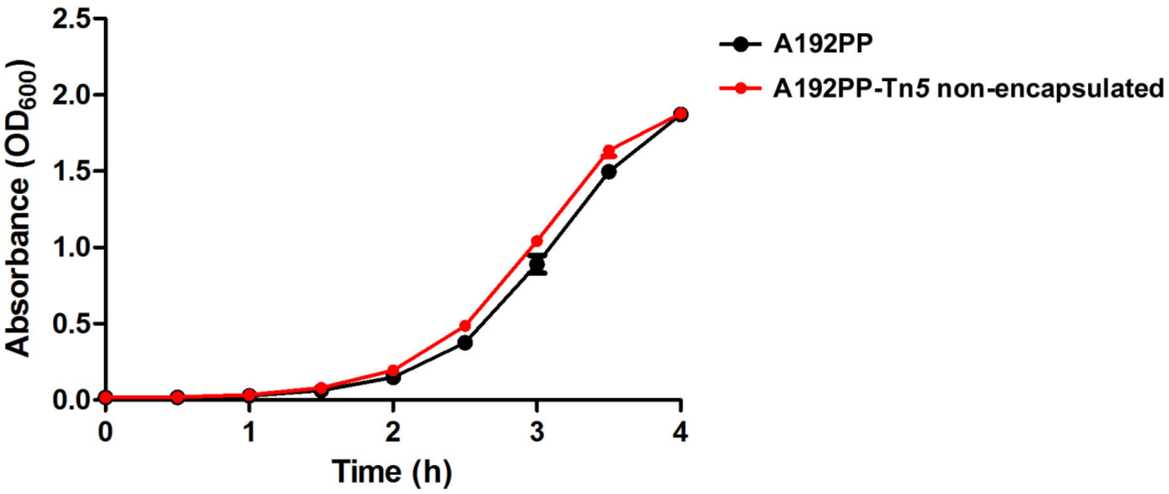

B

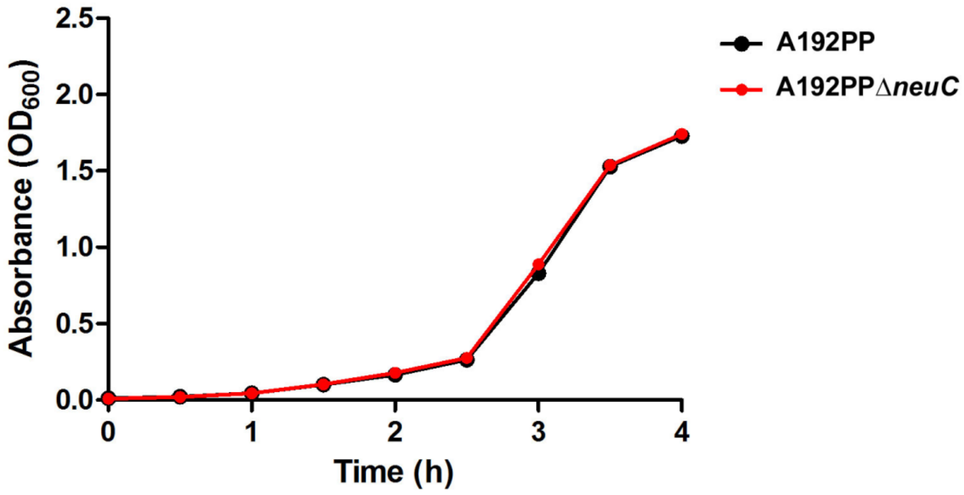

Strain	Mean Generation Time (min ± SD)
A192PP	24.45 ± 0.94
A192PP-Tn*5* non-capsulated	23.64 ± 0.21
A192PP	30.67 ± 0.52
A192PPΔ*neuC*	30.21 ± 0.941

FIG S2. Comparison of growth kinetics of a randomly selected non-encapsulated mutant from the Tn*5* TraDIS library in MH broth (A) and a non-encapsulated single gene mutant constructed using bacteriophage λ Red recombination in LB broth (B). *n*=3 in both cases. There were no significant differences in absorbance values at any time point when the log-rank [Mantel-Cox] test was applied. Student's *t* test was used to evaluate generation times.

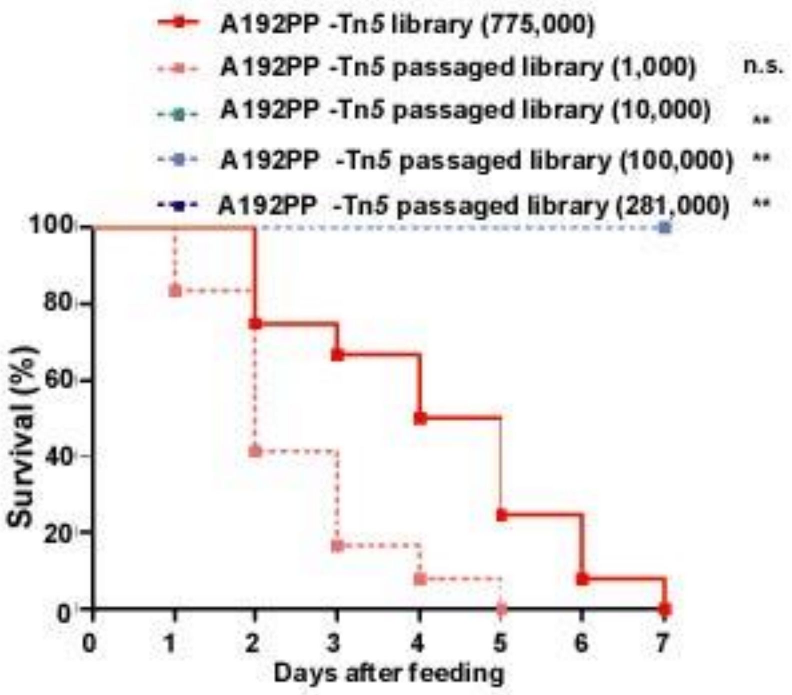

FIG S3. High-complexity cultured *E. coli* A192PP-Tn*5* libraries are avirulent in neonatal rats. Survival of P2 rats colonized with *E. coli* A192PP-Tn*5* libraries of differing complexities (1,000, 10,000, 100,000 or 281,000 mutants). Libraries were cultures in LB broth (8 h; 37°C) prior to initiation of colonization. Pups (n = 12 for each group) received 2-4x10^6 CFU by the oral route. Log-rank [Mantel-Cox] was used to compare rat survival following administration of cultured libraries with the uncultured complete library of 775,000 mutants: ns, non-significant, * $P < 0.05$, ** $P < 0.01$.

Table S4: Oligonucleotides for construction of targeted mutants

Gene	Primer	Sequence ($^{5'}$ to $^{3'}$)
lacZ	*lacZ*-P1	tggatttccttacgcgaaatacgggcagacatggcctgcccggttattatgtgtaggctggagctcttc
	lacZ-P2	tatgttgtgtgaaattgtgagcgaataacaatttcacacaggatacagctcatatgaatatcctccttag
neuC	*neuC-P1*	ctagagctgaatatggaatagttcggagacttttgacaatgctaagagaagtgtaggctggagctcttc
	neuC-P2	tgagaatcataacgaaagacaaaacaaagcactttttttctagtcataaccatatgaatatcctccttag
rfaH	*rfaH*-P1	cgtaaagcttttgctatccttgcgccccgattaaacggataagagtcattgtgtaggctggagctcttc
	rfaH-P2	ctggctgccaccacggatgccaatgtcaaaacactgtttgggattgcgttcatatgaatatcctccttag
traL	*traL*-P1	gtgaaatcctttcaattacaacctcctgtatttttccggcttcgcataaagtgtaggctggagctcttc
	traL-P2	cttatgataaataaaagtcgtcaaaattacaattacacggacatacaaaacatatgaatatcctccttag
vasL	*vasL*-P1	tctgcgtcatctcaaacagcaggagcgggcgtactgatggcaagtaacgcgtgtaggctggagctcttc
	vasL-P2	aggtcacatatcccttattggtacataaatcccccgatgtttactgacttcatatgaatatcctccttag
waaW	*waaW*-P1	atagtactcatccttaattattattgtaactcagacatccatgatttttagtgtaggctggagctcttc
	waaW-P2	taaaaaattaaaaggcaaagcgtaaaccacacagtcaaaacggaaccaaccatatgaatatcctccttag
yaeQ	*yaeQ*-P1	cgtattccgttacaatggcctcctgattcgaaaggagttttcttatggcgctgtgtaggctggagctcttc
	yaeQ-P2	actcgccatcagggatagcaacatgtcgggaaatcacaatcatgaaggttcatatgaatatcctccttag
yjiG	*yjiG*-P1	gccgatgaaatttcatcggcaactttgggcctttttagaaatggatttttgtgtaggctggagctcttc
	yjiG-P2	acaaatcattcctgttgtgattaatggtgatttcattatattcatcctgacatatgaatatcctccttag
0678	*0678*-P1	tagaaagtaaaattatcggacatttttatgccccacacagtgcattacccgtgtaggctggagctcttc
	0678-P2	aaggcgttgtatgccacacaacgcctcactgttcatttcttctttttctccatatgaatatcctccttag
3010	*3010*-P1	tcgcgaagaataatgatgaacttggcaaaggatatgattatgcgtattaagtgtaggctggagctcttc
	3010-P2	tatctataaacaaaaaccccatccggtgatttttgtcatttttttagccatcatatgaatatcctccttag

Table S5: Oligonucleotides for confirmation of targeted mutants

Gene	Primer	Sequence ($^{5'}$ to $^{3'}$)	Fragment size	
			wildtype	Δ
lacZ	*lacZ*-ampF	ATGCCGGTAATAATCCACAGC	3917	1600
	lacZ-ampR	TGCCATGTCCGGTTTTCAA		
neuC	*neuC*- ampF	GACAATGCCAGGAAAACAAG	1510	1600
	neuC- ampR	AAACGAAATAGCGGAGATTGT		
rfaH	*rfaH*- ampF	ACCACGGATGCCAATGTCA	664	1600
	rfaH- ampR	GTTCATCTTTGCGATGCTGT		
traL	*traL*- ampF	ACACGATTCTATTGGCCCTT	873	1600
	traL- ampR	GTATTTTTCCGGCTTCGCAT		
vasL	*vasL*- ampF	TCTGCCGGATCTCAGTCTGAT	1854	1600
	vasL- ampR	GGGCCACAGTCAAGAGGTTAA		
waaW	*waaW*- ampF	GGGTAATCATTGCTCATCGTG	1308	1600
	waaW- ampR	GGTAAAAGCTGTACGGCAGA		
yaeQ	*yaeQ*- ampF	AACTCTGTTTCGCAAGGTGA	771	1600
	yaeQ- ampR	AAAACGCAGATGAATAGCCG		
0678	*0678*- ampF	TGTCAGGGAGTGAAGAGACAA	705	1600
	0678- ampR	AAGTGCGTCGTTTACCGTCAT		
3010	*3010*- ampF	TTCTGTTCTAGATGCAAGGGC	318	1600
	3010- ampR	ATGATGAACTTGGCAAAGGA		
wzzE	*wzzE-ampF*	AAACGCAGACTGCGTAGAAA	1195	1600
	wzzE-ampR	GGCGCGTACCAAATACAGTCA		

Table S6: Oligonucleotides for construction of complemented mutants

Gene	Primer	Sequence ($^{5'}$ to $^{3'}$)
neuC	*neuC*-salI-F	CTAGTCGTCGACGACAATGCCAGGAAAACAAG
	neuC-sphI-R	GACTAGGCATGCAAACGAAATAGCGGAGATTGT
rfaH	*rfaH*-salI-F	CTAGTCGTCGACACCACGGATGCCAATGTCA
	rfaH-sphI-R	GACTAGGCATGCGTTCATCTTTGCGATGCTGT
traL	*traL*-sphI-F	GACTAGGCATGCACACGATTCTATTGGCCCTT
	traL-salI-R	CTAGTCGTCGACGTATTTTTCCGGCTTCGCAT
waaW	*waaW*-salI-F	CTAGTCGTCGACGGGTAATCATTGCTCATCGTG
	waaW-sphI-R	GACTAGGCATGCGGTAAAAGCTGTACGGCAGA
